# Absorption‐Dominant Electromagnetic Interference Shielding of Ti_3_C_2_T_x_ MXene‐Coated and Fe_3_O_4_‐Integrated TPMS Composites with Coupled Gradient Conductivity and Size‐Graded Structure

**DOI:** 10.1002/advs.202522856

**Published:** 2026-03-11

**Authors:** Abdallah Kamal, Baosong Li, Dawei Zhang, Muhammad Mujtaba Syed, Adam Otabil, Faisal Shahzad, Lianxi Zheng, Kin Liao

**Affiliations:** ^1^ Department of Mechanical and Nuclear Engineering Khalifa University of Science and Technology Abu Dhabi UAE; ^2^ Research & Innovation Center for Graphene and 2D Materials (RIC‐2D) Abu Dhabi UAE; ^3^ Department of Aerospace Engineering Khalifa University of Science and Technology Abu Dhabi UAE

**Keywords:** absorption‐dominant, EMI shielding, functionally graded structure, gradient coating, Ti_3_C_2_T_x_ MXene, TPMS Lattices

## Abstract

The increasing endeavor toward advanced electronic technologies leads to ubiquitous emission of electromagnetic waves (EMWs). To mitigate electromagnetic pollution, it is vital to develop absorption‐dominant EMI shields to stringently alleviate the secondary pollution caused by the highly conducting traditional shields. A functionally driven variable‐cell size and coating triply periodic minimal surface (TPMS) structure was created to realize stepwise longitudinal gradient impedance. Absorption‐dominant EMI shielding was achieved by coupling the intrinsic properties of Ti_3_C_2_T_x_ MXene coating with the benefits of a robust macrostructural graded TPMS structure. A gradient‐conductive MXene‐coated TPMS with a graded gyroid structure was fabricated via a hybrid process combining additive manufacturing and dip coating. In essence, the graded structure effectively orchestrates the journey of EMWs, first coaxing them inside and systematically draining its energy, resulting in superior EMI absorptivity within the X‐band range. Such a configuration endowed the structure with absorption‐dominant EMI shielding (absorptivity≈0.85) with an EMI SE_T_ of ≈ 59 dB, for 10 mm‐thick composites. Further increasing the thickness to 20 mm achieved a higher EMI SE_T_ of 68.07 dB with an extraordinary absorptivity of approximately 0.96. The developed design exhibited temperature insensitivity, affirming its environmental stability over a wide range of temperatures between −80°C to 200^°^C.

## Graphical abstract

A functionally driven variable‐cell size and gradient‐conductive coated TPMS structure was developed. Accordingly, stepwise longitudinal gradient impedance was realized, achieving absorption‐dominant EMI shielding (absorptivity≈0.85) with an EMI SE_T_ of ≈ 59 dB, for 10 mm‐thick composites. Further increasing the thickness of composites to 20 mm achieved a higher EMI SE_T_ of 68.07 dB with an extraordinary absorptivity of approximately 0.96.

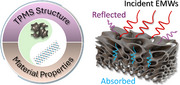

## 1 Introduction

Electromagnetic waves (EMWs) represent an indispensable foundation of the globally proliferated technologies, particularly in the aviation industries, communication, and radar systems [1]. Electromagnetic interference (EMI) shielding aims to block incident EMWs through reflection and absorption losses. This is essential to secure the reliable operation of the widespread sensitive electronic apparatus in telecommunications fields and mitigate their harmful consequences on human health [2]. Conventional EMI shielding materials, primarily highly conducting metals, provide rational performance; however, their high density, corrosion susceptibility, and excessive EMWs reflection restrict their implementation. Consequently, this escalates serious concerns about the inevitable exposure to EM secondary radiation pollution, entailing effective EMI shielding with high attenuation efficiency and absorption‐dominant capabilities [3].

2D materials constitute a prosperous class of nanomaterials that are atomically thin with an intrinsically sheet‐like morphology and a high surface‐to‐volume ratio, appealing mechanical, chemical, and electrical properties [4]. Graphene and MXenes are unique among 2D materials, and they stand out as promising candidates to engineer innovative broadband EMI shields and absorbers [5, 6]. MXenes are transition metal carbides, nitrides, or carbonitrides. They have a layered structure with a general formula of M_n+1_X_n_T_x_, where M represents an early transition metal (Ti, V, Nb, Mo), X can be carbon (C) and/or nitrogen (N), and T_x_ signifies surface terminations [7]. One of the most intriguing MXenes is Ti_3_C_2_T_x_, offering superb multispectral electromagnetic shielding capabilities [8]. This is derived from its excellent metallic electrical conductivity, which originates from the high charge carrier density (∼2 × 10^21^ cm^−3^) [9]. For instance, Rakhmanov et al. [10] unveiled that an ultrathin 4 nm‐thick Ti_3_C_2_T_x_ MXene film recorded an absorption‐dominant shielding with absorptivity (A) up to 0.5. Shahzad et al. [11] revealed that a freestanding 2.5 µm‐thick Ti_3_C_2_T_x_ film achieved a distinguished EMI SE of >50 dB. Nonetheless, it is hard to manipulate the shielding mechanism of such dense films, as they are typically dominated by dielectric loss and suffer from severe impedance mismatch due to their high electrical conductivity, leading to excessive reflection. Furthermore, freestanding films, foams, and aerogels made of MXene often experience worsened load‐bearing and mechanical scalability, hindering their full harness in real applications [12]. This necessitates the incorporation of Ti_3_C_2_T_x_ MXene into a matrix or loading it on a porous structure to pave the way for capturing its complete properties [13, 14]. For instance, Ti_3_C_2_T_x_ MXene and liquid metal‐integrated dual‐network hydrogels demonstrate multifunctional capabilities, including strain sensing and EMI shielding [15]. Additionally, the incorporation of magnetic Fe_3_O_4_ effectively compensates for some limitations by introducing magnetic loss mechanisms, improving impedance matching [16]. Fe_3_O_4_ nanoparticles suppress MXene restacking and generate abundant heterogeneous interfaces, which enhance interfacial polarization and multiple scattering effects [17]. As a result, the synergistic dielectric‐magnetic coupling in MXene/Fe_3_O_4_ composites enables absorption‐dominant, broadband EMI shielding with improved efficiency [18].

Additive manufacturing (AM) provides a feasible technology for creating adequately versatile designs for EMI shielding applications, overcoming the limitations of fabricating intricate structures [19, 20]. It enables precise tunability over the geometry and composition of the shields, realizing a synergy between the structure and the material attributes to customize the performance [21]. 3D printed graphene/polyamide‐6 composites with various thicknesses have been investigated to achieve reliable EMI shielding [22]. In parallel, metamaterials have arisen as tailorable designs for EM absorbers, where geometrical parameters can be finely customized to acquire a specific electromagnetic response [23, 24]. Parameterizable triply periodic minimal surface (TPMS) structures pose lightweight architectures with tunable porosity that enables impedance matching and enhanced structural absorption [25, 26]. Meanwhile, they generally possess mechanical integrity even at low densities. For instance, a TPMS structure composed of copper nanocube‐decorated polyurethane acrylate (Cu/PUA) was investigated for EMI shielding [27]. An et al. [28] demonstrated that the architectural parameters of TPMS structure, such as type, unit cell size, and relative density (RD), exert a pronounced influence on the EMI shielding response. Chemical deposition of Cu on a 6 mm‐thick PUA TPMS scaffold with a gyroid geometry effectively achieved a total EMI SE of 76.64 dB. Likewise, a robust Al_2_O_3_/SiO_2_ ceramic TPMS structure was successfully fabricated, showing a high compressive strength of 24 MPa and EMI SE of about 55 dB [29]. In another study, a SiOC ceramic gyroid TPMS structure with a thickness of 2.9 mm achieved a minimum reflection loss (RL_min_) of −59.96 dB [30]. SiC_w_@MXene/SiOC composites with gyroid TPMS structure (1.3–2.7 mm thick) showed an average EMI SE of 58.6–63.8 dB in the 0.2–1.6 THz range [31]. Integrating graphene in polymeric TPMS lattices has great potential to achieve outstanding EMI performance. For example, Polyvinylidene Fluoride (PVDF)‐graphene TPMS nanocomposites with various geometries accomplished specific shielding effectiveness (SSE) up to 94 dB cm^3^/g [32]. However, the performance exhibited high sensitivity toward several factors, including printing and lattice parameters.

Gradient‐conductive structures are another approach that shows tremendous significance for approaching high absorption. This gradual distribution of conductive material features a smooth impedance transition, yielding minimal reflection at the material‐air interface. For instance, hybridized MXene/reduced graphene oxide (rGO) aerogels established a gradient‐conductive profile, accomplished a RL_min_ of −60.1 dB [33]. Similarly, 3D‐printed gradient‐conductive MXene/CNT/Polyimide aerogel with hierarchical porosity demonstrated absorption‐dominant EMI shielding with an EMI SE of 68.2 dB and a reflectivity (R) of 0.23 [34]. An EMI SE of 65 dB with an absorptivity of 0.76 was realized by tuning the MXene content and the porosity level, achieving a gradient conductivity in a graded‐porosity MXene/polylactic acid (PLA) composite [35]. A compressed gyroid architecture composed of CNT/PLA showed an EMI SE of 67.0 dB while maintaining a Young's modulus of 4.43 GPa [36]. Wang et al. [37] reported that a 3D‐printed gradient‐conductive gyroid TPMS structure based on CNT‐coated polymeric scaffold attained an EMI SE of up to 35.9 dB with a reflectivity (R) of 0.13. Yao et al. [38] introduced an elegant strategy to engineer the absorption of a gyroid TPMS structure within X‐Ku band via gradually increasing the RD along the thickness, thereby delivering a longitudinal graded structure with commendable shielding performance.

Preserving EMI shielding performance is a burgeoning topic, particularly in harsh environments [39]. Pursuing efficient EMI shields with simultaneous temperature insensitivity is desirable to broaden their applicability in real‐world applications. Yao et al. [25] revealed that the absorption of a ceramic TPMS structure with a gyroid geometry is sensitive to temperature, where the RL_min_ enhanced from −58.05 to −72.38 dB after heating at 100^°^C. Another work proved that a graded gyroid TPMS structure made of ceramic material exhibited temperature insensitivity up to 500^°^C [38]. Ti_3_C_2_T_x_ MXene possesses high environmental stability over extended periods [5, 40]. Water‐ and oxidation‐resistant functionalized MXene exhibiting EMI SE of 52–77 dB was obtained, demonstrating high stability under elevated temperatures and 80% relative humidity for up to 49 days [41]. Yet, there are few studies on the influence of the operating temperature on the EMI shielding performance of 3D‐printed composites incorporating 2D materials.

In this work, the EMI shielding capabilities of gradient‐conductive Ti_3_C_2_T_x_ MXene‐coated graded gyroid TPMS structures were investigated. Initially, the influence of uniform unit cell sizes, ranging between 2.5 and 10 mm, on absorptivity was examined. The acquired results were then exploited to develop a graded TPMS structure aimed at minimizing reflection at the entry layer and facilitating deep penetration of the incident EMWs into the porous structure. To further enhance the attenuation of the waves, Fe_3_O_4_ nanoparticles were integrated into the printed graded lattices. The gradient‐conductive coating combined with a graded TPMS structure configuration exhibited absorption‐dominant EMI shielding, achieving absorptivity of ≈ 0.96 with an EMI SE of ≈ 68.07 dB. Finally, the mechanical properties and the environmental stability at various temperatures (−80°C to 200^°^C) were elucidated.

## 2 Experimental

### 2.1 Materials

Ti_3_AlC_2_ powder (MAX phase, particle size ≤ 40 µm) was obtained from Carbon‐Ukraine Ltd. (Ukraine). Isopropyl alcohol (C_3_H_8_O, 99%, IPA), lithium fluoride (LiF, 99%), and hydrochloric acid (HCl, 37%) were purchased from Sigma‐Aldrich (Germany). Commercial Fe_3_O_4_ particles (99.5%, 20 nm), shown in Figure S1, were procured from Macklin (China).

### 2.2 Synthesis of Ti_3_C_2_T_x_ MXene

Ti_3_C_2_T_x_ MXene was synthesized from Ti_3_AlC_2_ powder, applying the optimized minimally intensive layer delamination (MILD) method [42]. Briefly, the Ti_3_AlC_2_ powder was first prewashed with 9 M HCL (10 mL per 1 g Ti_3_AlC_2_). The pretreated powder was then collected and dried to remove impurities and excess Al. After that, an in situ hydrofluoric acid (HF) etchant was generated by mixing 1.6 g of LiF with 20 mL of 9 M HCl utilizing a Teflon‐coated magnetic stir bar in a high‐density polyethylene (HDPE) bottle at room temperature (RT) for 5 min. Afterward, 1 g of Ti_3_AlC_2_ (MAX phase) powder was slowly added to the etchant and stirred continuously at 400 rpm and 40°C for 30 h. The resulting sediment was washed with deionized (DI) water through several repeated centrifugation cycles at 3500 rpm for 10 min each. For redispersion, gentle hand shaking was applied. Washing was repeated until the supernatant reached pH ≈ 6. A stable Ti_3_C_2_T_x_ colloidal suspension was then obtained by centrifugation at 3500 rpm for 30 min, yielding a dark green dispersion with an adjusted Ti_3_C_2_T_x_ MXene concentration of 7.5 mg mL^−1^.

### 2.3 3D Printing of TPMS Structure

**FIGURE 1:**
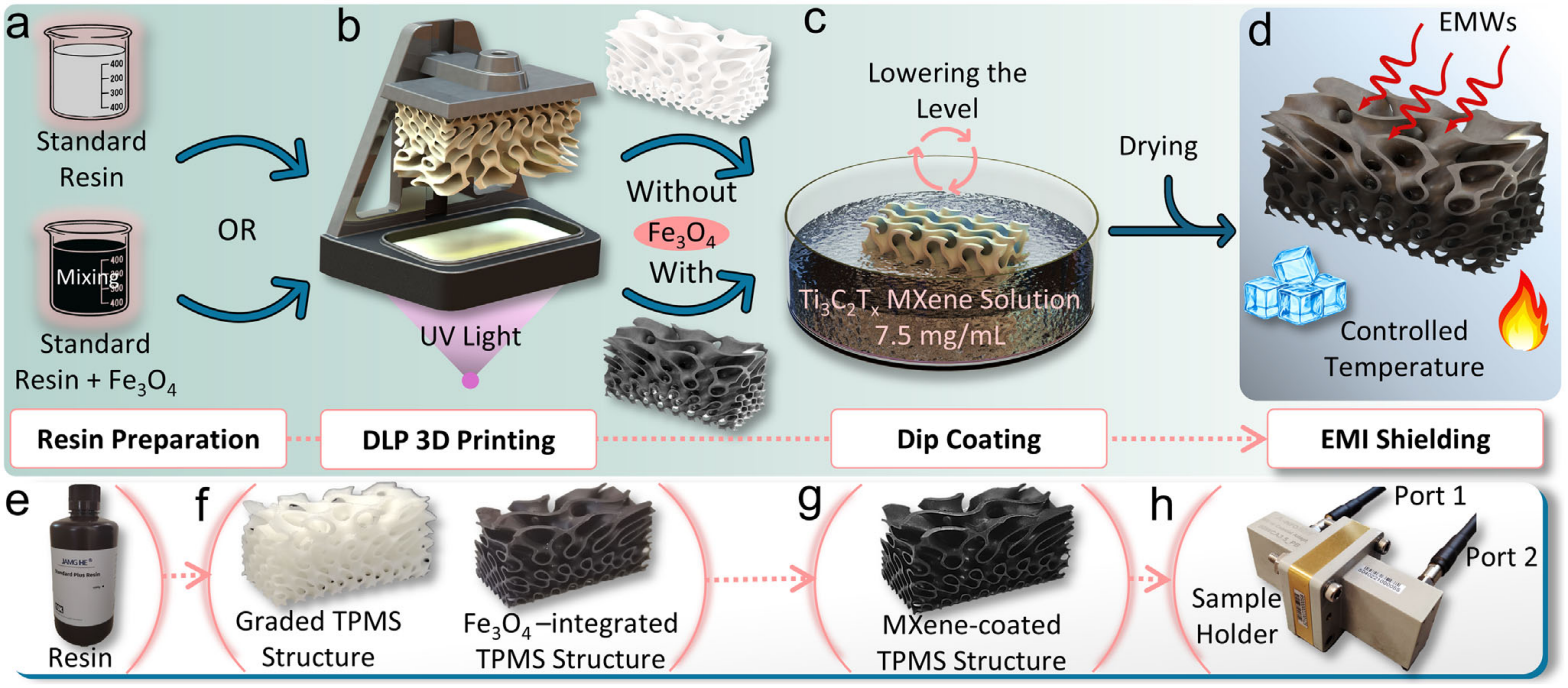
Schematic of the preparation procedure of the gradient‐conductive Ti_3_C_2_T_x_ MXene‐coated graded TPMS structures. (a) Preparation of the standard resin for printing. (b) Illustration of the DLP 3D printing technique. (c) Gradient‐conductive Ti_3_C_2_T_x_ MXene coating via dip coating approach. (d) EMI shielding measurement. (e) The utilized standard resin. (f) The printed graded TPMS structure without and with Fe_3_O_4_ magnetic particles. (g) Graded TPMS structure after Ti_3_C_2_T_x_ MXene coating. (h) Waveguide EMI shielding setup.

Digital light processing (DLP) 3D printing was employed to fabricate the TPMS structures. It offers one‐step fabrication of identical replicas of sophisticated 3D structures by selectively polymerizing photosensitive resins layer by layer. A DLP printer (Elegoo Saturn 4 Ultra 16K, 405 nm) supplied with standard resin (10K Plus White) was used to fabricate the gyroid TPMS samples. The printer features a high in‐plane XY resolution of 14 × 19 µm and a thin printing layer thickness of 15 µm, realizing accurate as‐designed TPMS structures with a trivial stair‐stepping effect. To fabricate the Fe_3_O_4_‐integrated TPMS samples, the standard resin was initially mixed with Fe_3_O_4_ magnetic powder at a controlled weight percentage (wt.%) using probe sonication for 2 h (Figure 1a). Concurrently, *MSlattice* software was utilized to model the TPMS geometries, which were then exported as STL files for slicing in *Chitubox* software [43]. Figure 1b illustrates the DLP printing method and the longitudinal graded TPMS structure. After printing, a two‐stage post‐treatment, including washing with isopropyl alcohol (IPA washing) and UV light exposure, was employed to remove residual uncured resin.

### 2.4 Fabrication of Ti_3_C_2_T_x_ MXene‐Coated and Fe_3_O_4_‐Integrated TPMS Structures

The preparation procedure of the EMI shielding TPMS structures is schematically illustrated in Figure 1. Briefly, after printing the TPMS lattices and preparation of Ti_3_C_2_T MXene solution, the fabricated structures with and without Fe_3_O_4_ magnetic powder were dip‐coated in Ti_3_C_2_T_x_ MXene solution with a concentration of 7.5 mg/mL at RT. The dip coating process was conducted for 30 min under vacuum to ensure good impregnation and uniform MXene coating with tight adhesion, as shown in Figure 1c. To achieve the longitudinal gradient conductive coating, the MXene solution level was lowered after each immersion cycle. First, the entire structure was fully immersed in the MXene solution. After drying, it underwent a partial immersion by reducing the liquid level to represent two‐thirds of the sample thickness. Lastly, the solution level was lowered to cover only the bottom one‐third of the sample. This stepwise immersion approach yielded a 10 mm‐thick gradient‐conductive composite, with the top, middle, and bottom layers (≈3.33 mm each) coated once, twice, and three times, respectively. However, to obtain uniformly coated lattices, it was maintained at the same level to completely cover all lattices. Then all lattices were vacuum‐dried at 60^°^C for 3 h after each coating cycle to remove confined water. This technique guarantees effective MXene deposition on the lattice's surface in a controlled manner. The obtained gyroid gradient‐conductive Ti_3_C_2_T_x_ MXene‐coated with Fe_3_O_4_‐integrated TPMS lattices were ingeniously fabricated, as shown in Figure 1e–h.

### 2.5 Characterization

PANalytical Empyrean X‐ray diffractometer was used to obtain X‐ray diffraction (XRD) patterns, employing Cu‐Kα radiation (λ = 0.1542 nm) at a voltage and current of 45 kV and 40 mA, respectively. A field‐emission scanning electron microscope (JEOL 7610, Japan) was used to capture high‐magnification images of Ti_3_AlC_2_ particles, Ti_3_C_2_T_x_ MXene flakes, the cross‐sectional area of the Ti_3_C_2_T_x_ film, the surface morphology, and the energy dispersive spectrum (EDS) of the fabricated Ti_3_C_2_T_x_ MXene‐coated TPMS structures. X‐ray photoelectron spectroscopy (XPS) was performed using an ESCALAB Xi^+^ X‐ray photoelectron spectrometer. An Ossila Four‐Point Probe System (MCP‐T610 model, Mitsubishi Chemical, Japan) was used to measure the electrical conductivity of the TPMS structure's surface. Compression and cyclic loading tests were carried out utilizing an Instron universal testing machine (Instron 5948 UTM) equipped with a 100 N load cell. For compression tests, cubic TPMS samples were placed between two parallel plates and loaded at a constant strain rate of 0.1 min^−1^. The average value of the results of five tested samples was reported.  Thermogravimetric analysis (TGA) of the TPMS lattices, under N_2_ atmosphere, was conducted by a Simultaneous Thermal Analyzer‐STA 449 F5 Jupiter machine.

### 2.6 Electromagnetic Interference Shielding Measurement

A 2‐port vector network analyzer (Rohde & Schwarz ZNA43, 10MHz‐43.5 GHz) equipped with a WR‐90 rectangular waveguide was utilized for measuring EMI shielding, the relative complex permittivity (ε_r_ = ε_r_′‐jε_r_″), and the relative complex permeability (µ_r_ = µ_r_′‐jµ_r_″) over the X‐band region (8.2–12.4 GHz). Rectangular samples with a 22.86 × 10.16 mm^2^ size were employed for the measurements to fit the EMI sample holder. The power coefficients of absorptivity (A), reflectivity (R), and transmissivity (T) were calculated from the measured complex scattering (S) parameters S_11_, S_21_, S_22_, and S_12_ using Equations ((1), (2), (3)), respectively. These power coefficients are endorsed to compare the contribution of reflection and absorption to the overall shielding capability. Moreover, electromagnetic interference shielding effectiveness (EMI SE) is a vital quantitative index to estimate the shielding performance. The reflection shielding effectiveness (SE_R_) and absorption shielding effectiveness (SE_A_) can be calculated using Equations (4) and (5), respectively. Then the total EMI shielding effectiveness (SE_T_) is expressed in Equations (6) and (7) [44].

(1) $$R={\left|S_{11}\right|}^{2}={\left|S_{22}\right|}^{2}$$

(2) $$T={\left|S_{21}\right|}^{2}={\left|S_{12}\right|}^{2}$$

(3) A=1−T−R

(4) $$SE_{R}\left(dB\right)=-10\;\log\;\left(1-R\right)$$

(5) $$SE_{A}\left(dB\right)=-10\;\log\left(\frac{T}{1-R}\right)$$

(6) $$SE_{T}\left(dB\right)=-10\;\log\left(T\right)$$

(7) $$SE_{T}=SE_{A}+SE_{R}$$

### 2.7 Statistical Analysis

The EMI shielding properties and mechanical properties data are expressed as the mean ± standard deviation (SD), based on 4–6 replicates for each test.

## 3 Results and Discussion

### 3.1 Characterization of Ti_3_C_2_T_x_ MXene

**FIGURE 2:**
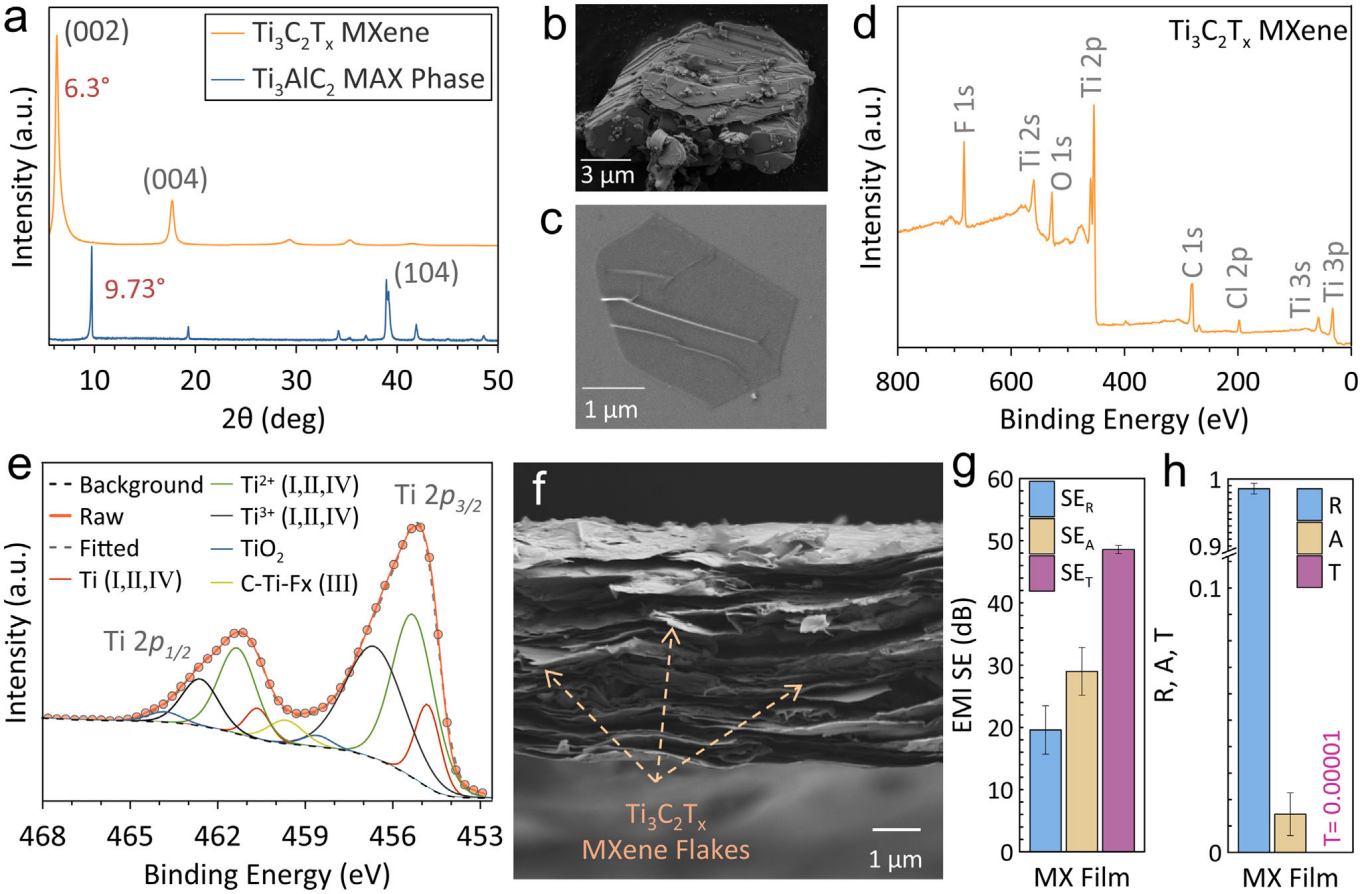
Characterization of the as‐synthesized Ti_3_C_2_T_x_ MXene. (a) XRD patterns of the Ti_3_AlC_2_ MAX phase powder and the Ti_3_C_2_T_x_ MXene film prepared. SEM image of (b) Ti_3_AlC_2_ MAX phase particles and (c) Ti_3_C_2_T_x_ MXene monolayer on a silicon substrate. (d) Survey XPS spectrum of Ti_3_C_2_T_x_ MXene. (e) Deconvoluted peaks of the high‐resolution XPS spectrum of Ti 2p. (f) Cross‐sectional SEM image of the Ti_3_C_2_T_x_ MXene film. EMI shielding performance of Ti_3_C_2_T_x_ MXene film (g) EMI SE and (h) Reflectivity, absorptivity, and transmissivity.

Ti_3_C_2_T_x_ MXene stands out as a prominent 2D material with superior EMI shielding performance, comparable to that of metals even at the nanoscale [45]. Their exceptional electrical conductivity and rich surface chemistry enable the formation of highly conductive coatings on diverse polymeric substrates [46]. Ti_3_C_2_T_x_ MXene is generally synthesized via a wet chemical etching approach, endowing it with abundant surface functional groups such as ─F, ─OH, and ═O. The complete etching of the interleaved Al layers within the Ti_3_AlC_2_ MAX phase and the subsequent exfoliation of Ti_3_C_2_T_x_ MXene layers were verified by XRD analysis, as evidenced by a noticeable shift of the characteristic peak of (002) plane to a lower 2θ value (6.3°) after etching, indicating an increased interlayer spacing [47, 48]. The vanishment of the (104) peak (Figure 2a) further verifies the successful etching process [49]. As shown in Figure 2b, the SEM image displays the bulk structure of the Ti_3_AlC_2_ MAX phase, whereas the exfoliated Ti_3_C_2_T_x_ MXene exhibits a thin 2D morphology with lateral dimensions of approximately 3 µm (Figure 2c). Moreover, the XPS spectrum displays the key elements Ti and C without residual Al (Figure 2d), confirming the successful Al layer etching. The deconvoluted components of the high‐resolution XPS spectrum of the Ti 2p region (Figure 2e) affirm the coexistence of Ti‐C bonds. This further corroborates the synthesis of Ti_3_C_2_T_x_ MXene with typical functionalized surfaces, which is consistent with earlier studies [50].

Assembling Ti_3_C_2_T_x_ MXene nanoflakes via the vacuum‐assisted filtration (VAF) process yields a dense film with a hierarchically aligned structure with precisely controlled thickness [12]. Initially, a pure 4µm‐thick MXene film with a weight of ≈ 6 mg was fabricated to evaluate its EMI shielding performance. The MXene film shows a compact layered structure constructed of tightly overlapped MXene flakes, as observed from its cross‐section (Figure 2f). Serving as EMI shields, the Ti_3_C_2_T_x_ MXene film exhibits remarkable SE_R_, SE_A_, and SE_T_ of about 19.58±3.9, 28.99±3.84, and 48.58±0.67 dB, respectively, as presented in Figure 2g. Noticeably, a reflection‐dominant shielding is observed for the Ti_3_C_2_T_x_ MXene film, evidenced by a high power coefficient of reflectivity (R) of 0.985 (Figure 2h). This performance is owing to its high electrical conductivity (σ = 2911 ± 82 S cm^−1^), arising from the dense and compacted structure of the Ti_3_C_2_T_x_ MXene film. This maximizes the impedance mismatch at the air‐film interface, promoting the reflection of the incident radiation. Herein, redistributing a comparable quantity of Ti_3_C_2_T_x_ MXene across a 3D architecture, such as TPMS lattice frameworks, induces a transition toward absorption‐dominant shielding while sustaining efficient overall EMI SE.

### 3.2 Gradient Conductive Ti_3_C_2_T_x_ MXene‐Coated TPMS With Uniform Structure

**FIGURE 3:**
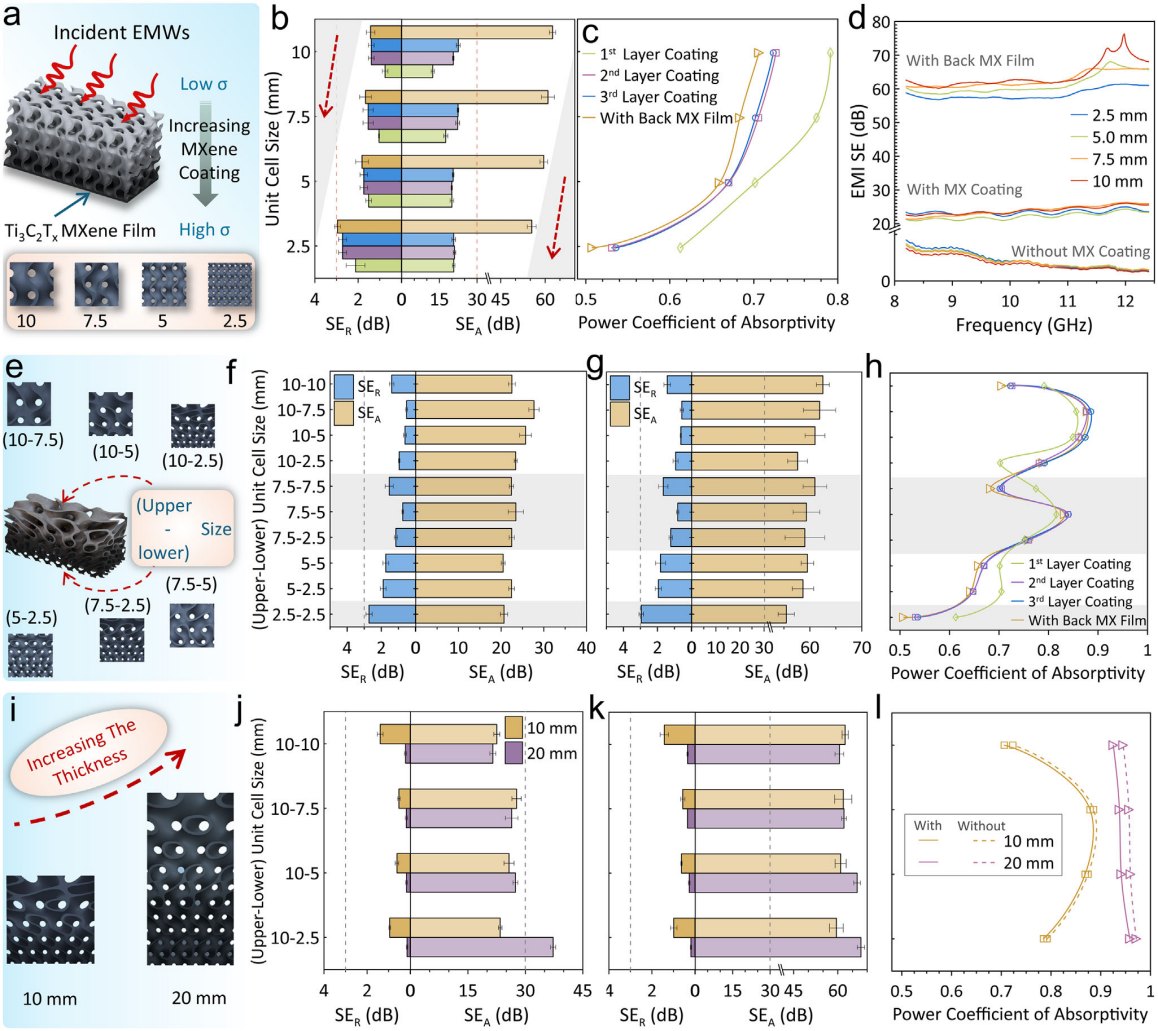
(a) Illustration of the gradient‐conductive coating on a uniform TPMS structure. (b) SE_A_ and SE_R_, (c) Absorptivity in terms of unit cell size, and (d) EMI SE_T_ of gradient‐conductive Ti_3_C_2_T_x_ MXene‐coated TPMS with uniform gyroid structure. (e) Illustration of the gradient‐conductive gyroid TPMS with graded structure. SE_A_ and SE_R_ in terms of gradient size (f) Without and (g) With back Ti_3_C_2_T_x_ MXene film. (h) Absorptivity as a function of the degree of gradient size. (i) Schematic illustration of a functionally graded TPMS structure with various thicknesses. The Effect of sample thickness on SE_A_ and SE_R_ of TPMS structure with various gradient size (j) Without and (k) with back Ti_3_C_2_T_x_ MXene film. (l) Absorptivity in terms of sample thickness.

Typically, absorption‐dominant EMI shields feature a multilayered configuration with impedance‐matching, absorption, and reflection layers [51]. The entry layer (incident end) should possess relatively low electrical conductivity, which reduces impedance mismatch at the air‐material interface, thereby retaining minimal front‐surface reflection and allowing the incident EMWs to penetrate the structure and reach the subsequent layers. The absorptive layer should be engineered with lossy dielectric components to disrupt the propagation and convert the transmitted EM energy into heat through dielectric and magnetic losses. The rear reflective layer, positioned at the outgoing end, is highly conductive, reflecting any residual EMWs back into the absorptive region. This secondary pass stimulates further attenuation, thereby boosting the total shielding efficiency [52]. As schematically illustrated in Figure 3a, such a configuration encourages EM waves to enter, undergo absorption, and be attenuated, rather than merely bouncing off the surface. By tailoring the electrical conductivity of the layers into a gradual gradient rather than forming sharp interfaces, the shield achieves a smoother impedance transition from free space into the interior of the material. This gradient‐conductive architecture enhances wave penetration depth, reduces reflection loss, and promotes efficient conversion of EM energy into heat.

To regulate the EMI shielding performance, the influence of the unit cell size of TPMS lattice frameworks was systematically examined. Gyroid sheet‐based TPMS structures with four different unit cell sizes (2.5, 5, 7.5, and 10 mm) and a fixed RD of 20% were coated with an identical gradient‐conductive Ti_3_C_2_T_x_ MXene layer, as displayed in Figure 3a. The selected sizes are smaller than the operating wavelength of the X‐band to establish effective structural dissipation. Additionally, the sheet‐gyroid TPMS structure was chosen due to its higher absorptivity that originates from its larger porosity and surface area compared to the strut‐gyroid type [28, 53]. The gradient‐conductive Ti_3_C_2_T_x_ MXene coating was achieved by sequentially lowering the MXene solution level by one‐third of the sample height after each immersion cycle. Evidently, the EMI SE_A_ of the composite increases with the addition of successive coating layers, as shown in Figure 3b. The improvement is particularly pronounced in samples with larger unit cell sizes. The unit cell size influences markedly both the overall EMI SE and the underlying shielding mechanism. With an increase in stepwise MXene coating, the composite demonstrates a slight decrease in absorptivity (A), as depicted in Figure 3c. Notably, all composites exhibit absorption‐dominant shielding, where all of them achieved A > 0.5 and SE_R_ < 3 dB. In addition, a larger unit cell size leads to higher absorptivity and EMI SE. Specifically, the EMI SE gradually shifts from high SE_A_ and low SE_R_ to lower SE_A_ and higher SE_R_ as the size of the unit cell decreases from 10 to 2.5 mm, as depicted in Figure 3b. Correspondingly, the absorptivity dropped markedly from 0.723 ± 0.028 for the 10 mm unit cell to 0.536 ± 0.004 for the 2.5 mm unit cell, as shown in Figure 3c. Such behavior is independent of the amount of coated Ti_3_C_2_T_x_ MXene (coating cycles), aligning well with earlier studies [35, 53]. Additionally, the unit cell size exerts a minimal influence on the SE_T_, which remained around ≈23 dB.

Placing a 4 µm‐thick pure Ti_3_C_2_T_x_ MXene film on the highly conductive side of the structure had a significant impact on the EMI shielding performance. The highly reflective back Ti_3_C_2_T_x_ MXene film (R = 0.985) (Figure 2h) drastically boosted the EMI SE_T_ of the lattices to ≈ 60 dB. However, the absorptivity decreased marginally by less than 0.5% of those without the back film. The highly conductive Ti_3_C_2_T_x_ film traps the EMWs by reflecting the transmitted radiation back to be absorbed and attenuated by the structure via multiple scattering. Figure 3d shows the EMI SE_T_ as a function of frequency for different unit cell sizes. The high absorptivity of the TPMS structure with a larger unit cell size is ascribed to its larger pores, which facilitate EM wave transmission into the interior regions and motivate the formation of magnetic ring cavities. On the other hand, smaller sizes predominantly trigger the losses through the induced currents [28].

### 3.3 Gradient Conductive Ti_3_C_2_T_x_ MXene‐Coated TPMS With Graded Structure

Varying the unit cell size of the TPMS structure across its thickness, creating a graded structure, allows for availing a wide combination of pore sizes and enables customized EMI shielding behavior. To explore the graded‐size effect, diversiform samples with distinct combinations of upper and lower unit cell sizes were fabricated, as depicted in Figure 3e, where the assigned first and last numbers indicate the unit cell size at the upper and lower surfaces, respectively. For instance, the sample labeled 10–2.5 corresponds to a graded gyroid structure sample with a large 10 mm unit cell at the entry layer (upper surface) and a smaller 2.5 mm unit cell at the shielding layer (lower surface). Accordingly, it possesses a large pore size at the entry layer, which progressively decreases toward the lower surface, ending with a smaller pore size. Interestingly, introducing a trivial size gradient drastically boosts the acquired EMI SE_A_ and suppresses EMI SE_R_, compared with the uniform structures having the same entry unit cell size. Moreover, EMI SE_R_ of the coated samples gradually increased with the degree of cell size gradient, as presented in Figure 3f. The EMI SE_A_ upsurged considerably from 22.53 ± 0.723 dB for the uniform 10‐10 sample to 27.71 ± 1.22 dB for the mildly graded 10‐7.5 sample, then gradually decreased to 25.724 ± 1.3 dB and 23.41 ± 0.47 dB for the more strongly graded 10‐5 and 10‐2.5 samples, respectively. Conversely, EMI SE_R_ accomplished 0.523 ± 0.057 dB, 0.613 ± 0.082 dB, and 0.95 ± 0.0042 dB for 10‐7.5, 10‐5, and 10‐2.5 samples, respectively. Meanwhile, the absorptivity (A) increased vividly to 0.886±0.013 for the 10‐7.5 sample, an enhancement of 22.5% over the uniform 10‐10 structure. Such improvement slightly declined with increasing size gradient, reaching 0.874±0.009 and 0.792±0.02 for 10‐5 and 10‐2.5 samples, as presented in Figure 3h. These observations substantiate the presence of a strongly absorption‐dominant shielding mechanism in all graded samples. Pursuing high‐performance EMI SE, the gradient samples developed were placed in front of a highly conductive pure Ti_3_C_2_T_x_ MXene film. Notably, this configuration achieved EMI SE ≈ 60 dB while maintaining the characteristic absorption‐dominant shielding mechanism of the gradient TPMS structures, indicating effective reflection–reabsorption synergy, as shown in Figure 3g,h.

To gain further insight into the influence of cell size grading, the EMI shielding of the cell size‐graded structure with different thicknesses of 10 and 20 mm was evaluated. Compared with the 10 mm‐thick structures, the 20 mm‐thick samples achieved substantially higher EMI SE values accompanied by very low SE_R_ (<0.25 dB), as expected. Moreover, the SE_A_ increases, and the SE_R_ decreases with an increase in cell size grading, as shown in Figure 3j. The 10‐2.5 samples achieved 37.24±0.7 dB, which represents about 59% higher than that of the 10 mm‐thick samples. The same upward trend is maintained after introducing the back MXene film, where the SE_T_ reaches 68.07±1.24 dB for 10‐2.5 samples, as shown in Figure 3k. Interestingly, a notable enhancement in absorptivity is observed for the 20 mm‐thick sample (A>0.945), which further increased to 0.971 for the highly graded 10‐2.5 structure, as presented in Figure 3i. Introducing the back MXene film caused only a minor reduction (∼1.5%) in absorptivity (A). These results demonstrate a strong correlation between thickness and the degree of grading, enabling effective tuning of absorption performance. The thicker samples have a smoother transition from the larger unit cell size to a smaller size, generating a wider distribution of pore sizes and interfacial area, as presented in Figure 3i. Additionally, this provides more interaction surface and a more elegant impedance transition, which in turn leads to enhanced absorption [38].

### 3.4 Gradient Conductive Ti_3_C_2_T_x_ MXene‐Coated and Fe_3_O_4_‐Integrated TPMS Graded Structure

**FIGURE 4:**
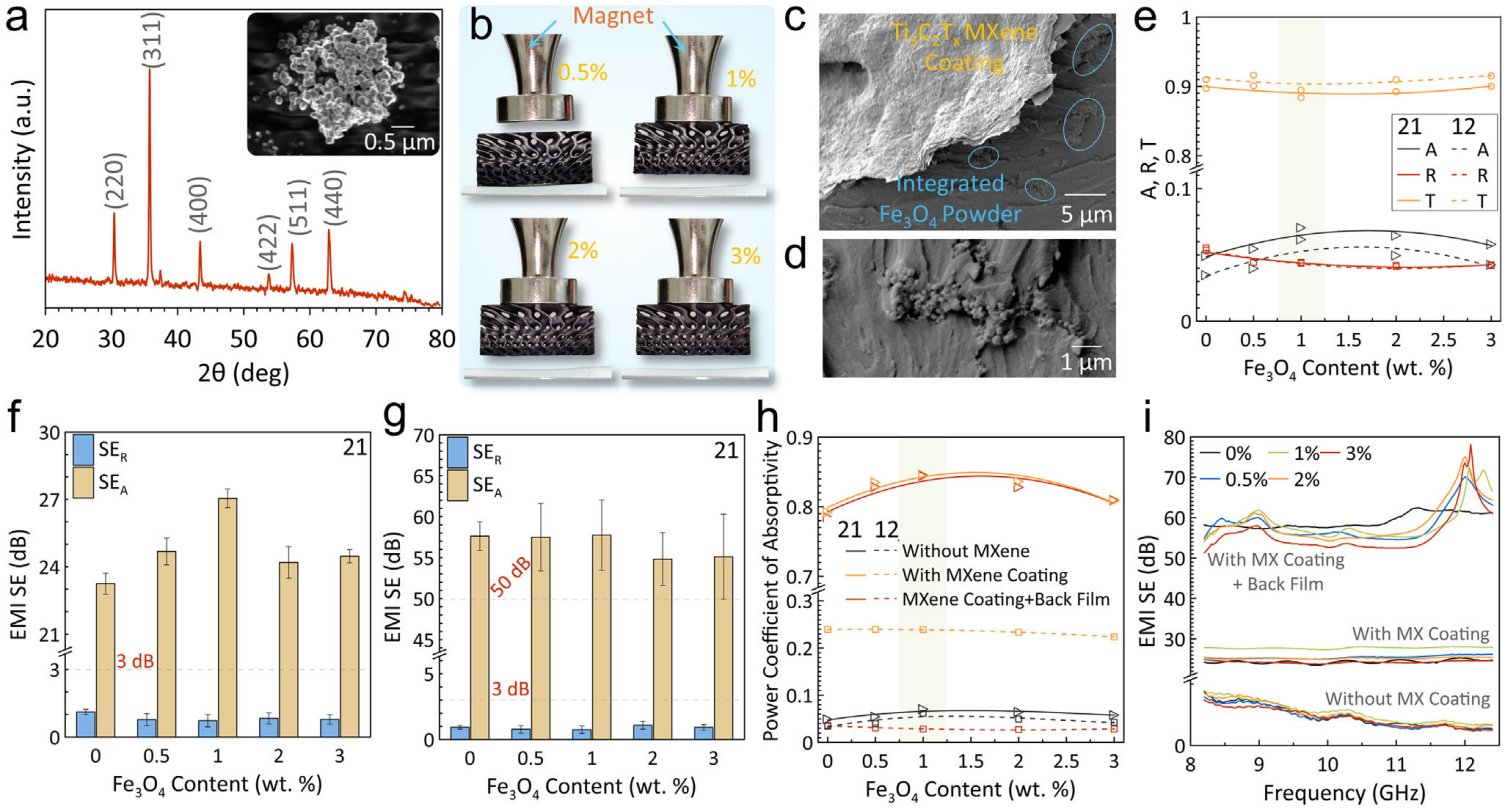
Characterization of the utilized Fe_3_O_4_ nanoparticles (a) XRD pattern and (inset: SEM image). (b) Images of the as‐fabricated samples under a magnetic bar. SEM images of (c) The fabricated gyroid gradient‐conductive Ti_3_C_2_T_x_ MXene‐coated with Fe_3_O_4_‐integrated TPMS lattices, (d) Magnified morphology of the integrated Fe_3_O_4_ particles. EMI shielding of Fe_3_O_4_ integrated‐TPMS structure (e) Absorptivity in terms of various Fe_3_O_4_ content. Effect of Fe_3_O_4_ weight percentage on the EMI SE of samples (f) with Ti_3_C_2_T_x_ MXene coating and (g) with both Ti_3_C_2_T_x_ MXene coating and back film in the 21 direction. (h) Effect of Fe_3_O_4_ weight percentage on the absorptivity. (i) Total EMI SE versus frequency in terms of Fe_3_O_4_ weight percentage in the 21 direction.

Incorporating Fe_3_O_4_ magnetic particles as an auxiliary filler into the printed graded TPMS structure bestows additional synergistic magnetic losses, bringing magnetic‐dielectric coupling that enhances the attenuation of EMWs. To fabricate magnetic‐dielectric hybrid architectures, Fe_3_O_4_ magnetic nanoparticles (Figure 4a) were uniformly mixed with the standard resin at controlled weight fractions prior to 3D printing. Unfortunately, the DLP‐based 3D printing of Fe_3_O_4_‐embedded composites is constrained by light attenuation, as high Fe_3_O_4_ contents lead to strong absorption and scattering of the curing light, hindering resin polymerization. However, graded TPMS structures (sample 10‐2.5) incorporating up to 3 wt.% Fe_3_O_4_ was successfully fabricated, as presented in Figure 4b. SEM images (Figure 4c,d; Figure S3) show uniform Ti_3_C_2_T_x_ MXene coating with good adhesion on Fe_3_O_4_‐embedded TPMS structures.

To isolate the influence of Fe_3_O_4_, the EMI shielding performance of the Fe_3_O_4_‐integrated TPMS structures without Ti_3_C_2_T_x_ MXene coating was first examined. As illustrated in Figure 4e, the structures exhibited weak attenuation, where most EMWs are transmitted (A < 0.1 and T > 0.88). Likewise, the EMI SE appears to be low, where both SE_R_ and SE_A_ are less than 0.5 dB, as shown in Figure S2. However, increasing the Fe_3_O_4_ loading improved the shielding performance, and at 1 wt.% Fe_3_O_4_, both EMI SE_A_ and absorptivity (A) peaked at 0.335 dB and 0.071, respectively. Further increases in the Fe_3_O_4_ loading deteriorate the performance, delivering 0.273 dB and 0.058, respectively, at 3 wt.%. Upon coating with gradient‐conductive Ti_3_C_2_T_x_ MXene, a similar performance trend was sustained. The EMI SE_A_ improves by approximately 15.5%, and the SE_R_ is decreased by 23.5%, achieving 27.05 and 0.727 dB, respectively, for the 1 wt.% Fe_3_O_4_‐loaded sample compared with the neat resin, as displayed in Figure 4f. Concurrently, the absorptivity (A) improves, reaching 0.846 ± 0.052, which is equivalent to 106.8% of that of the neat structure (Figure 4h), reflecting the synergistic effect of magnetic and conductive losses. To further modify the EMI SE, a pure Ti_3_C_2_T_x_ MXene film is attached to the TPMS structure on the highly conductive end. The EMI SE_T_ in Figure 4g increases drastically to 58.5 ± 4.2 dB for the 1 wt.% Fe_3_O_4_ sample. This enhancement was accompanied by a negligible reduction in absorptivity (<0.5%), which achieved 0.844, as shown in Figure 4h. Adding 1 wt.% Fe_3_O_4_ increases the absorptivity (A) by about 7.5% compared with the neat samples. The variation of EMI SE_T_ with frequency and Fe_3_O_4_ weight percentage is summarized in Figure 4i.

The incidence direction plays a decisive role in gradient‐conductive TPMS structures, governing the prevailing shielding mechanism through asymmetric impedance matching. Comparison of EMI shielding results obtained from the 21 direction (based on S_11_ and S_21_) and the 12 direction (based on S_22_ and S_12_) clearly demonstrates that the graded architectures are sensitive to the direction of the incoming EMWs. That dependency primarily originates from the intrinsic properties of the entry layer facing the EMW source, such as the porosity and the electrical conductivity [52]. Specifically, in the 21 direction, the larger 10 mm unit cell faces port 1, and the smaller 2.5 mm unit cell faces port 2. Conversely, in the 12 direction, this arrangement is reversed, with the 2.5 mm unit cell facing port 1 and the 10 mm unit cell facing port 2, as illustrated in Figure S2. The variation in the EMI SE and absorptivity derived from different incident directions of the Fe_3_O_4_‐integrated samples with and without Ti_3_C_2_T_x_ MXene coating further reinforces the effect of the unit cell size, as presented in Figure S2. Clearly, when the EMWs impinge on the smaller unit cell surface (12 direction), the EMI SE_A_ and absorptivity (A) are notably reduced relative to the 21 direction, as shown in Figure 4e. However, this directional effect became more pronounced after introducing the gradient‐conductive Ti_3_C_2_T_x_ MXene coating, where the size effect is coupled with the level of electrical conductivity. In the 12 direction, the smaller unit cell (2.5 mm) with higher conductivity facing the incident EMWs exhibits stronger reflection and lower absorption compared to the 21 direction, where the larger unit cell (10 mm) and less conductive surface are exposed. Consequently, absorption‐dominant and reflection‐dominant EMI shielding behaviors were discerned along the 21 and 12 directions, respectively. This trend was consistently observed across all investigated Fe_3_O_4_ weight percentages. Moreover, Figure S2 shows the EMI SE in the 12 direction when a back Ti_3_C_2_T_x_ MXene film is applied, which exhibits nearly identical performance to that of the pure MXene film.

**FIGURE 5:**
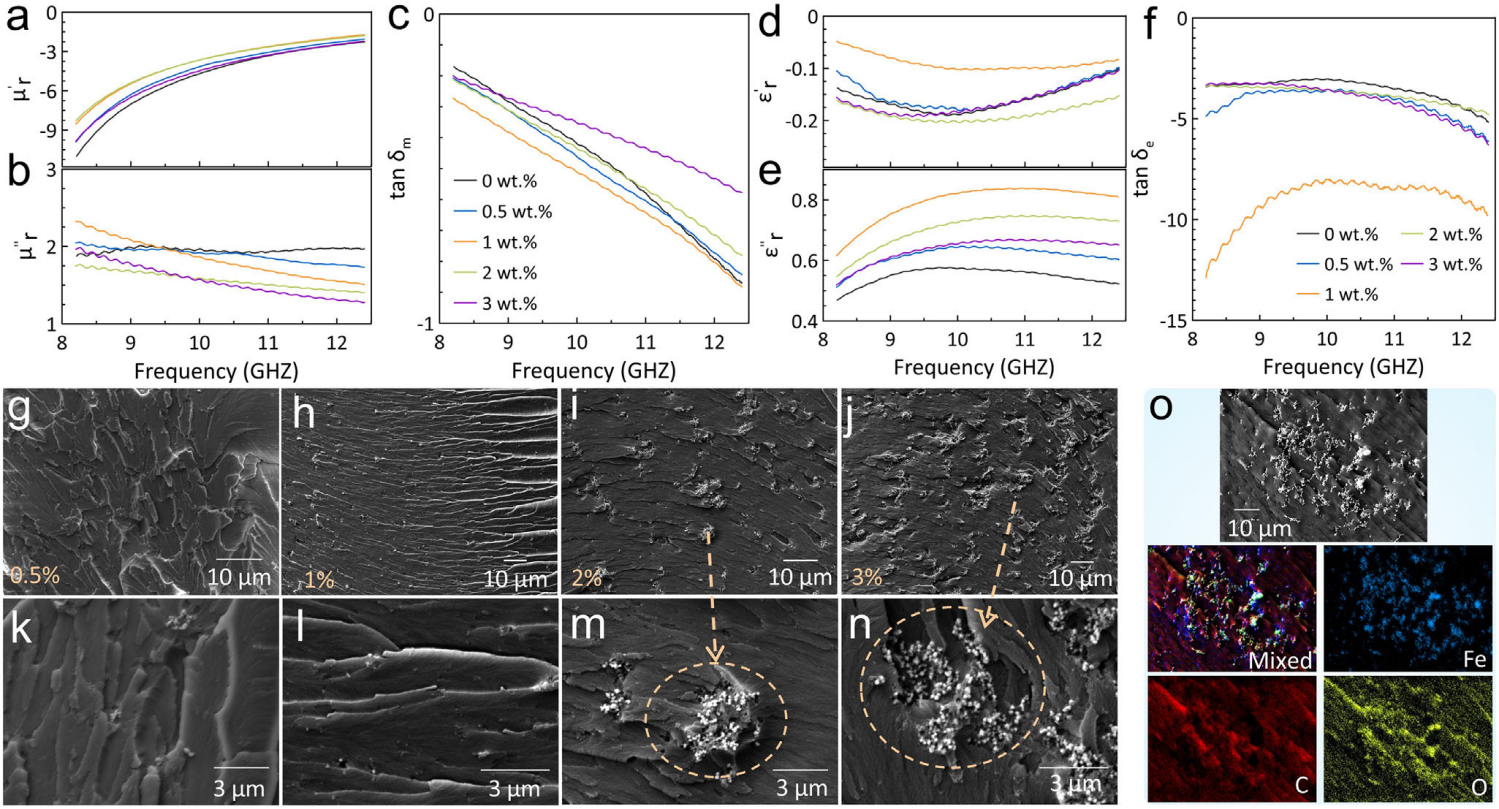
Electromagnetic parameters of the acquired Fe_3_O_4_‐integrated TPMS graded structure. (a) Real permeability, (b) Imaginary permeability, (c) Magnetic tangent loss, (d) Real permittivity, (e) Imaginary permittivity, and (f) Electrical tangent loss. SEM images of the gradient‐conductive Ti_3_C_2_T_x_ MXene‐coated with (g–k) 0.5 wt.%, (h–l) 1 wt.%, (i–m) 2 wt.%, and (j–n) 3 wt.% Fe_3_O_4_‐integrated TPMS Structure. (o) EDS Elemental mapping of 2 wt.% samples.

The permittivity and permeability (ε_r_ and µ_r_) of the shield material exclusively affect the EMI shielding efficiency. 2D Ti_3_C_2_T_x_ MXene is an intrinsically non‐magnetic material that exhibits a relative permeability (µ_r_) ≈ 1, contributing minimal magnetic losses. Nevertheless, its shielding behavior primarily arises from dielectric and conductive losses. Figure 5a–f shows the electromagnetic properties of the TPMS structure exhibiting deliberately tailored dielectric/magnetic properties. Notably, the developed TPMS structure exhibited simultaneously negative real permittivity (ε_r_′) and permeability (µ_r_′) components, manifesting obvious features of the left‐handed metamaterials that hold tremendous significance as EM absorbers [54, 55]. The incorporation of Fe_3_O_4_ magnetic particles into the printed polymeric lattices significantly enhanced the ε_r_ and µ_r_ of the system, strengthening the impedance matching and the absorption via magnetic losses. Major improvements were noted for the MXene‐coated samples, mainly resulting from the multiple scattering actions induced by the highly conductive internal surfaces. Obviously, the EM parameters of 1 wt.% Fe_3_O_4_‐integrated samples surpass those of other loading levels. Embedding 1 wt.% Fe_3_O_4_ confers the lattices with superior energy storage and dissipation capabilities. This likely arises from the homogeneous distribution of Fe_3_O_4_ particles throughout the lattices, as shown in Figure 5g–j. However, the inferior performance of higher Fe_3_O_4_ contents (2 and 3 wt.%) is due to the agglomeration of Fe_3_O_4_ particles, as demonstrated in Figure 5k–n and Figure S4. EDS elemental analysis of the internal region of the fabricated TPMS structure and an agglomeration of Fe_3_O_4_ in a 3D‐printed sample with 2 wt.% are shown in Figure S5 and Figure 5o.

### 3.5 EMI Shielding Mechanisms

**FIGURE 6:**
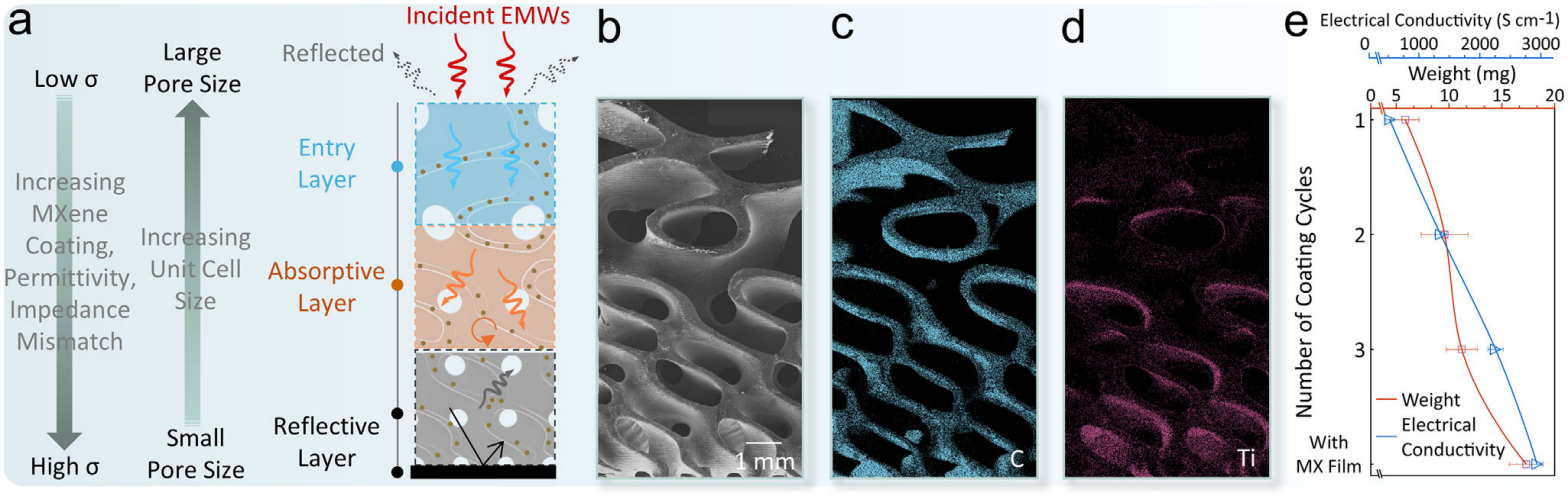
(a) Schematic illustration of the proposed gradient design. (b) SEM image of the graded TPMS structure. EDS elemental analysis of the gradient‐conductive Ti_3_C_2_T_x_ MXene‐coated TPMS graded structure (c) C and (d) Ti. (e) The weight of the loaded Ti_3_C_2_T_x_ MXene and the subsequent electrical conductivity after each coating cycle.

The gyroid TPMS structure is a binary composite material of air and the substrate material with tortuous interpenetrating channels and adjustable porosity, as shown in Figure 6a, offering highly tunable EMI shielding capabilities. SEM image (Figure 6b) clearly demonstrates a smooth and continuous morphological transition across the graded structure. As anticipated, the EDS elemental distribution of Ti_3_C_2_T_x_ MXene increases along the thickness with a pronounced accumulation at the bottom, constructing continuous conductive pathways, as revealed in Figure 6c,d. When EMWs impinge on the shield, their energy is partitioned into reflected, absorbed, and transmitted components. The low‐conductive entry layer with large pores ensures minimum impedance mismatch that allows the EMWs to enormously penetrate the structure, achieving minimal reflection. Meanwhile, it partially contributes to absorbing and attenuating the waves. According to the simplified Fresnel Equation (8), SE_R_ is tightly related to the electrical conductivity and the shield material's permeability [56]. Reducing the impedance mismatch by minimizing the electrical conductivity of the entry layer and increasing the permeability drastically reduces interface discontinuities and enhances the absorptivity as depicted in Equation (9).

(8) $$SE_{R}\left(dB\right)=20\;\log\left[{\left(Z+Z_{o}\right)}^{2}/4ZZ_{o}\right]=39.5+10\;\log\left(\sigma /2\pi \mu f\right)$$

(9) $$SE_{A}\left(dB\right)=8.69d\;{\left(\pi f\mu \sigma \right)}^{0.5}$$

(10) $$\delta =\sqrt{2/\omega \mu \sigma }$$

where Z_o_ and Z are the impedances of free space and the shield, respectively. σ is the electrical conductivity (S m^−1^), *µ* is the magnetic permeability (H m^−1^), *d* is the thickness in m, *f* is the electromagnetic frequency (Hz), and *ω* is the angular frequency (rad/s). Then, a stepwise increment of the impedance mismatch within the intermediate dissipation layer by increasing the electrical conductivity and reducing the pore size induces excessive multiple reflections, as shown in Figure 6e. During the multiple scattering actions, the confined EMWs are progressively attenuated since increasing the electrical conductivity along with the magnetic permeability reduces the skin depth (*δ*), improving the absorption and the decay of the EMWs' energy, as expressed in the foregoing formula (10). This effect is further magnified by the large interacting surface area and the elongated propagation paths. The penetrated EMWs are eventually suppressed by the bottom highly conductive shielding layer, where they become trapped, repeatedly reflected, and fully dissipated within the dissipation layer.

The overall dissipation mechanism hinges on two primary avenues, encompassing material dissipation and structure dissipation [57]. The attenuation of the EMWs is attributed to the dielectric and magnetic losses in the Ti_3_C_2_T_x_ MXene and Fe_3_O_4_ particles, respectively, where the EMW energy dissipates into heat [58]. The dielectric losses of Ti_3_C_2_T_x_ MXene arise from both ohmic and polarization losses. The ohmic loss originates from the movement of free electrons in the conductive Ti_3_C_2_T_x_ MXene network [59]. Polarization loss is initiated by the abundant functional groups of the Ti_3_C_2_T_x_ MXene and the interfacial interactions between Ti_3_C_2_T_x_ MXene and the coated polymer, which construct nanoscale dipole polarization and microscale interfacial polarization, respectively [60]. Such an effect is increasingly prominent due to multiple reflections within the Ti_3_C_2_T_x_ MXene layers. Furthermore, the magnetic loss induced by Fe_3_O_4_ particles is associated with the behavior of the electrons in the *d* orbitals, involving alignment and realignment due to the oscillating magnetic field. Such loss mechanisms substantially aid in alleviating and dissipating the alternating electric and magnetic fields. Whereas structural dissipation is predominantly related to the spatial distribution and the geometrical configuration of the graded architecture. Gyroid TPMS geometry features distinct spherical voids that trigger the formation of current‐loop cavities, inducing local magnetic fields and magnetic resonance [53].

### 3.6 Thermal Stability

**FIGURE 7:**
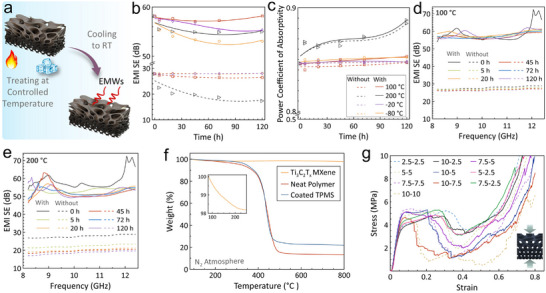
(a) Schematic illustration of preparing and testing the EMI samples. EMI shielding performance in terms of temperature and duration of 1 wt.% Fe_3_O_4_ ‐integrated TPMS gradient structure. (b) Total EMI SE. (c) Absorptivity. Total EMI SE versus frequency at (d) 100°C and (e) 200°C. (f) TGA curves of the prepared MXene, neat polymeric structure, and MXene‐coated TPMS structures. (g) Stress–strain curves of the gradient‐conductive Ti_3_C_2_T_x_ MXene‐coated TPMS with a uniform and graded structure.

Utilization of the TPMS graded structure in extreme environments with varying temperatures is vital to expand its implementation as a multifunctional design. Ti_3_C_2_T_x_ MXene is renowned for its comparatively high thermal stability among MXenes family [61]. To verify the stability of the obtained structure, their EMI shielding performance was evaluated after exposure to various temperatures in air, ranging from −80°C to 200^°^C, for different durations up to 120 h, as shown in Figure 7a–c. The measured total EMI SE and absorptivity (A) exhibited negligible variation after thermal exposure at 100^°^C up to 120 h (Figure 7d), confirming the structural and electrical stability of the MXene‐based composite. This behavior is primarily attributed to the inherent chemical and thermal stability of MXene between 25°C and 100^°^C. However, at 200^°^C, there is a drop in the measured total EMI SE, which is accompanied by an increase in the absorptivity level. The total EMI SE stabilized at about 55 dB after 5 h, as shown in Figure 7e. However, for cryogenic conditions, the TPMS structure maintained extraordinary stability even after exposure to −80^°^C for 120 h. The reduction of the total EMI SE after the thermal treatment stems from the evaporation of the confined water molecules and the partial loss of the surface functional groups, leading to a reduced interlayer spacing [12, 62]. Increasing the surface electrical conductivity densifies the reflection, guiding the wave toward the opposite end of the structure. These changes increase the charge‐carrier mobility and density (electrons) due to the removal of electron scattering sites such as surface functional groups [45]. Consequently, the samples heated at 200^°^C exhibit higher absorptivity as a result of improved electrical conductivity and reduced interfacial scattering. TGA analysis of a neat Ti_3_C_2_T_x_ MXene film (Figure 7f) exhibits high thermal stability up to 800^°^C. However, minor weight loss (about 2%) is seen at approximately 150°C due to evaporation of the trapped water molecules. The neat polymeric scaffold shows stability up to about 300^°^C, followed by a sharp weight loss between 350°C–500^°^C due to the decomposition of the polymer. Interestingly, coated TPMS structures have enhanced thermal stability compared to that of the neat one, which is accompanied by higher residual weight (about 20%–25%) at 800^°^C. To emphasize the long‐term stability of the coated samples, the EMI shielding performance of the samples was first reevaluated after three months of storage at ambient temperature. The results show a slight reduction in both EMI SE_T_ and absorptivity (A), as shown in Figure S6. Then, the long‐term EMI shielding stability after storage in a high‐humidity (95%) environment for ten days was evaluated. The samples demonstrated high stability with a small reduction in the EMI shielding performance, confirming the chemical stability of the Ti_3_C_2_T_x_ MXene coating.

### 3.7 Mechanical Properties

Gyroid TPMS architecture is a distinct structure that is known for its mechanical integrity, effectively balancing low density with high stiffness over other counterparts for structural applications [63]. To ensure the load prerequisites of the structure during service, their mechanical performance and deformation behavior in terms of unit cell size and graded size degree were evaluated under compressive loading. Figure 7g represents the disparities in the obtained stress‐strain curves of the TPMS structure. It is evident that they exhibit comparable stress‐strain curves with minor variations in the acquired mechanical properties. The obtained yielding stress values vary between ≈ 3.3 and 5 MPa. Before the yield point, at a strain of ≈ 6%, the stress‐strain curves feature linear elastic characteristics. After the yield point, the curves display a distinct plateau feature with a subtle softening across a strain range of ≈ 6%–25%, depending on the unit cell size and degree of grading. Beyond that, a sharp collapse and subsequent upward densification at a strain of ≈ 40% were observed, consistent with previously reported deformation behavior of TPMS structures [37]. The unit cell size of the uniform TPMS structures has a prominent impact on the mechanical characteristics, as presented in Figure S7. Apparently, the yield strength is prone to enhancement with increasing the unit cell size. However, the TPMS structure with a 10 mm unit cell size deviated from the upward trend, having a lower yield point with the lowest failure strain. Additionally, structures with a smaller unit cell size (2.5 mm) display an expanded plateau phase with gradual collapse, featuring uniform deformation due to uniform stress distribution. In contrast, increasing the unit cell size induces a shorter plateau stage, sharp local collapse, and notable fluctuations due to the localized stresses, causing enormous structural damage. The graded TPMS structure exhibits smooth stress transfer over the structure, retaining similar mechanical characteristics of the small‐sized cell structures [64]. For instance, the 10‐2.5, 7.5‐2.5, and 5‐2.5 samples manifested mechanical behavior comparable to the uniform structure with a 2.5 mm unit cell size, further remarkably affirming the robustness exemplified by the graded structure. Additionally, as shown in Figure S8, the designed structure exhibits stable mechanical performance of over 1000 loading cycles without any signs of structural collapse when subjected to cyclic loading at a stress level just below the yield point (5% strain).

## 4 Conclusions

Embodying the synergy between material and structure to attain efficient EMI shielding requires painstaking efforts. The effective interplay between the constituent materials and the TPMS architecture significantly dictates the overall EMI shielding attributes. Establishing such a configuration effectively alleviates the impedance mismatch at the incident side, facilitating deep penetration of the EMWs and reducing the reflection. The porous architecture subsequently disrupts wave propagation and promotes the conversion of EM energy into thermal dissipation, resulting in superior EMI absorptivity. In this work, absorption‐dominant EMI shielding TPMS structures were successfully fabricated. Coupling a gradient‐conductive coating of Ti_3_C_2_T_x_ MXene on a graded gyroid TPMS structure integrated with Fe_3_O_4_ particles accomplished an absorptivity up to 0.85 with a total EMI SE of approximately 59 dB for 10 mm‐thick samples. Increasing the thickness to 20 mm further enhanced absorptivity to about 0.96 and total EMI SE of 68.07 dB, which is significant in the context of obtaining absorption‐dominant shielding materials. Moreover, the developed design exhibited excellent mechanical integrity and environmental stability between −80°C to 200°C while preserving reliable EMI shielding performance.

## Funding

The authors appreciate the support provided by the Research & Innovation Center for Graphene and 2D Materials (RIC‐2D) of Khalifa University under Award No. DP4‐8434000508.

## Conflicts of Interest

The authors declare no conflicts of interest.

## Supporting information




**Supporting File**: advs74273‐sup‐0001‐SuppMat.docx.

## Data Availability

The data that support the findings of this study are available in the supplementary material of this article.
