# Supplementary material for: Absorption‐Dominant Electromagnetic Interference Shielding of Ti3C2Tx MXene‐Coated and Fe3O4‐Integrated TPMS Composites with Coupled Gradient Conductivity and Size‐Graded Structure
Source: Adv Sci (Weinh). 2026 Mar 11;13(22):e22856. doi: 10.1002/advs.202522856 (PMC13088346; doi:10.1002/advs.202522856)
Supplement: Supplementary file 1 — Supporting File: advs74273‐sup‐0001‐SuppMat.docx. [file ADVS-13-e22856-s001.docx]

**Absorption-Dominant Electromagnetic Interference Shielding of Ti_3_C_2_T_x_ MXene-coated and Fe_3_O_4_-integrated TPMS Composites with Coupled Gradient Conductivity and Size-Graded Structure**

Abdallah Kamal^1,3^, Baosong Li^2,3^, Dawei Zhang^1^, Muhammad Mujtaba Syed^1,3^, Adam Otabil^1,3^, Faisal Shahzad^3^, Lianxi Zheng^1,3^, Kin Liao^2,3,*^

1. Department of Mechanical and Nuclear Engineering, Khalifa University of Science and Technology, 127788, Abu Dhabi, UAE.

2. Department of Aerospace Engineering, Khalifa University of Science and Technology, 127788, Abu Dhabi, UAE.

3. Research & Innovation Center for Graphene and 2D Materials (RIC-2D), 127788, Abu Dhabi, 9 United Arab Emirates.

- **Corresponding Authors**

Prof. Kin Liao ([kin.liao@ku.ac.ae](mailto:kin.liao@ku.ac.ae))

**Absorption-Dominant Electromagnetic Interference Shielding of Ti_3_C_2_T_x_ MXene-coated and Fe_3_O_4_-integrated TPMS Composites with Coupled Gradient Conductivity and Size-Graded Structure**

1. **Fe_3_O_4_ Nanoparticles**

Commercial Fe_3_O_4_ particles (99.5%, 20 nm), shown in **Fig. S1**, were procured from Macklin (China) and used in this study.


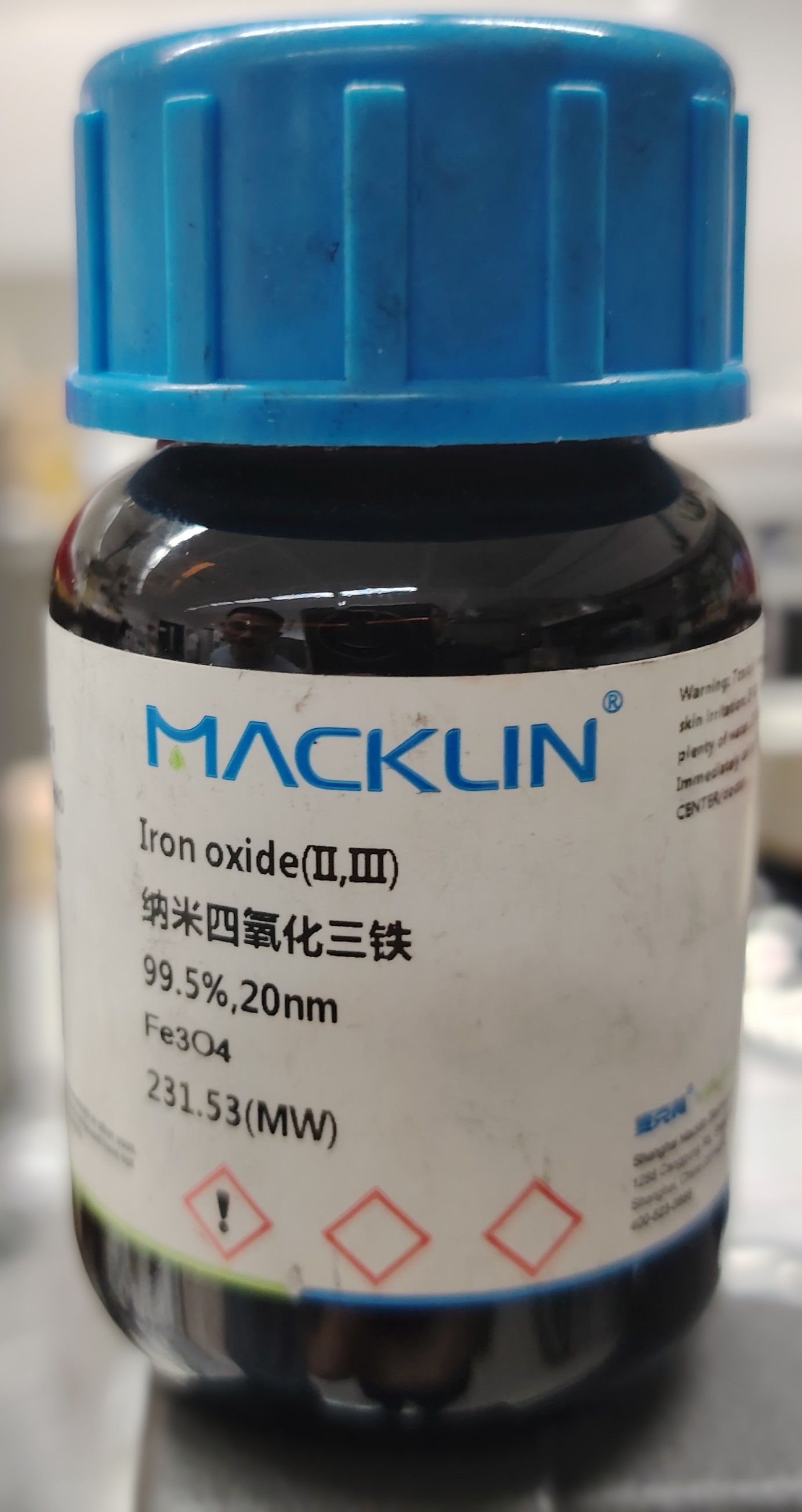


**Fig. S1** Fe_3_O_4_ nanoparticles used in this study.

1. **EMI Shielding Measurement**


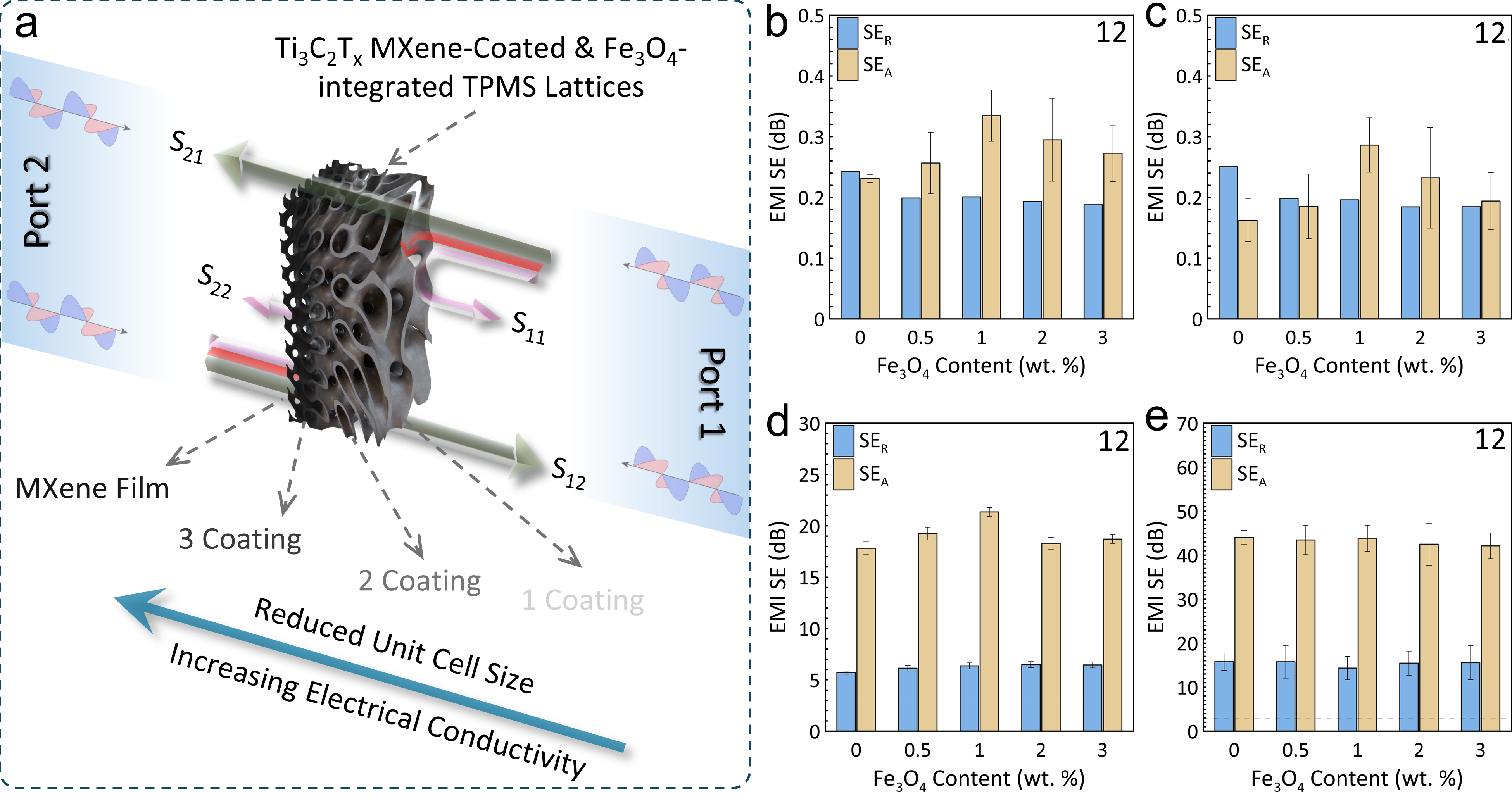


**Fig. S2** Effect of Fe_3_O_4_ weight percentage on the EMI SE of samples without Ti_3_C_2_T_x_ MXene coating in **a** 21 and **b** 12 directions. Effect of Fe_3_O_4_ weight percentage on the EMI SE of samples **c** with Ti_3_C_2_T_x_ MXene coating and **d** with Ti_3_C_2_T_x_ MXene back film in 12 direction.


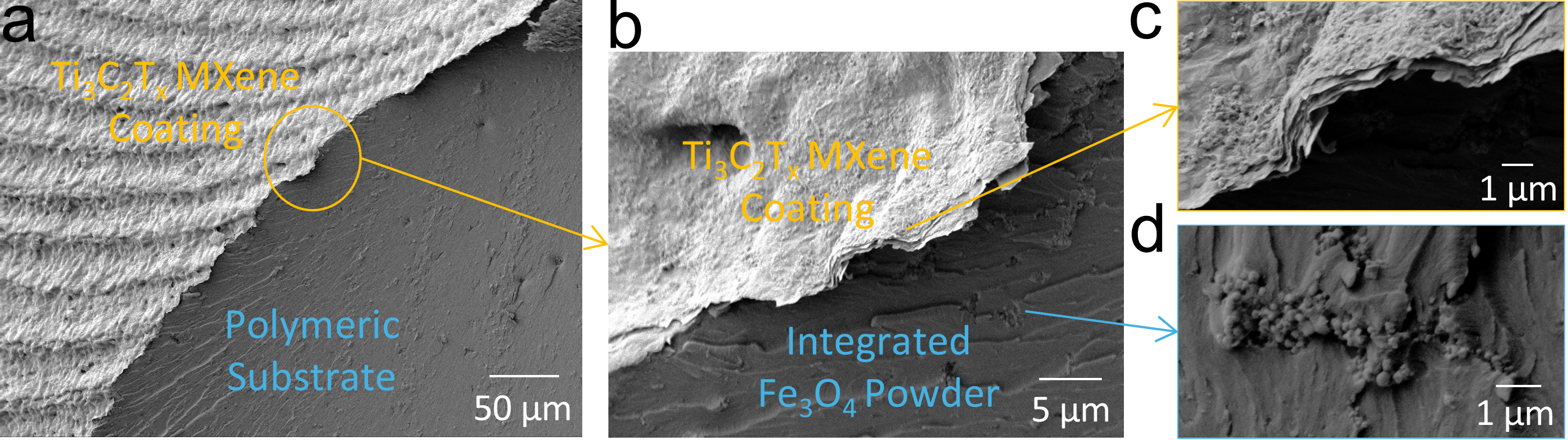


**Fig. S3** SEM images of **a,b** The fabricated gyroid gradient-conductive Ti_3_C_2_T_x_ MXene-coated with Fe_3_O_4_-integrated TPMS lattices, **c** Ti_3_C_2_T_x_ MXene coating layers, and **d** Magnified morphology of the integrated Fe_3_O_4_ particles.


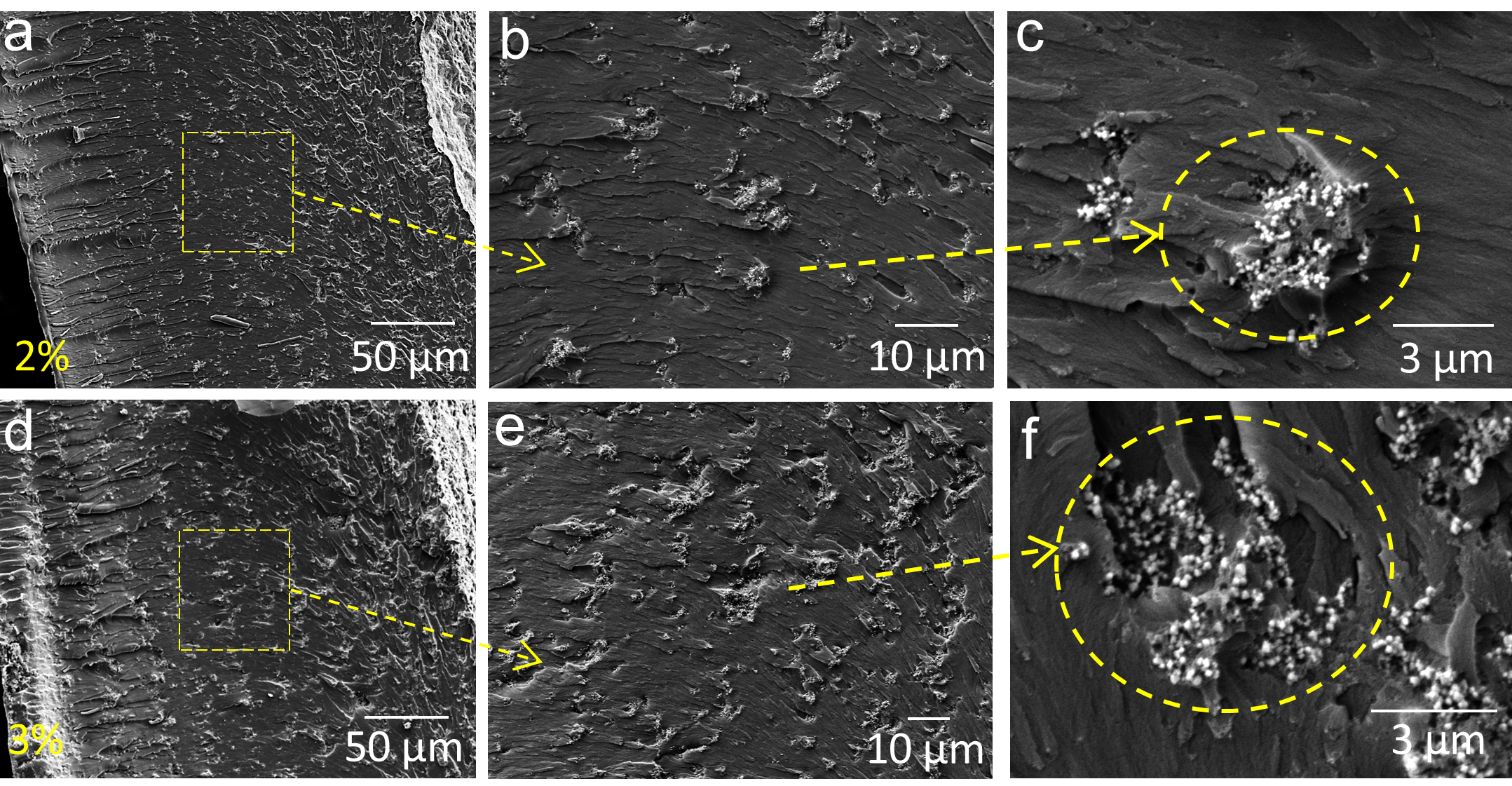


**Fig. S4** SEM images of the fabricated gyroid gradient conductive Ti_3_C_2_T_x_ MXene-coated with **a,b,c** 2 wt.% **d,e,f** 3 wt.% Fe_3_O_4_-integrated TPMS lattices from large to small scale.


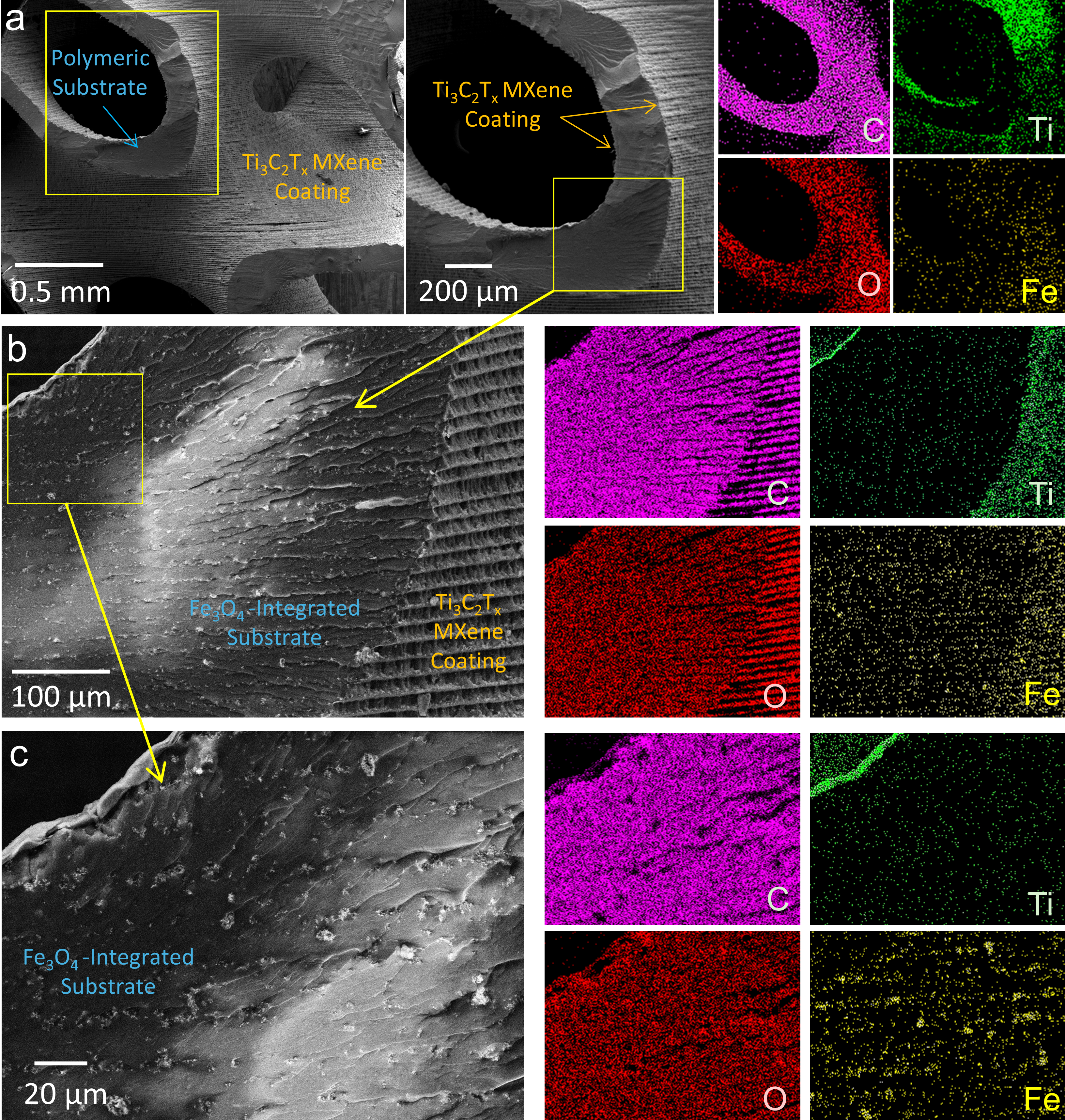


**Fig. S5** EDS elemental mapping analysis of the internal region of the fabricated gyroid gradient conductive Ti_3_C_2_T_x_ MXene-coated with 2 wt.% Fe_3_O_4_ nanoparticles from large to small scale.

1. **Long-Term Stability**

Notably, Ti_3_C_2_T_x_ MXene exhibits long-term chemical stability for 5 years of storage under ambient conditions, if it is stored in a solid state where trivial water content has existed. As reported by Lee et al. [18], Ti₃C₂Tₓ MXene synthesized via the MILD approach and subsequently formed into dense films, shows significantly suppressed oxidation and degradation. The reduced interlayer spacing in dense MXene layers effectively limits the ingress of air and water, thereby preventing oxidation of Ti₃C₂Tₓ MXene, as shown in **section 3.4**.

Additionally, previous work done by our research group [19] demonstrated that Ti₃C₂Tₓ MXene stacks maintain excellent chemical and mechanical stability up to 200 °C, consistent with reports from other studies [20],[21]. Consequently, the EMI shielding performance remains highly stable even after thermal treatment at 200 °C for 5 days, as shown in **Fig. 7**.


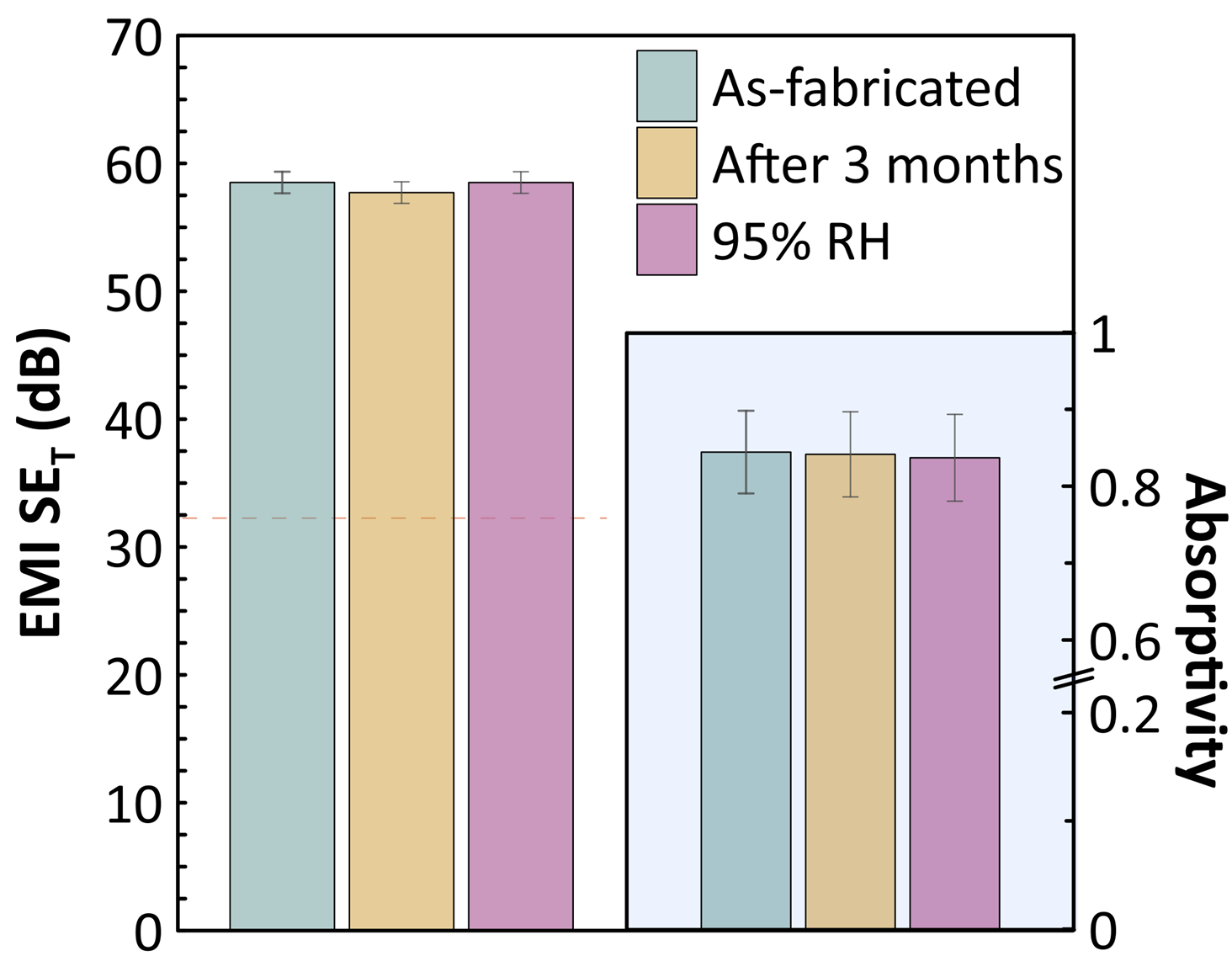


**Fig. S6** Total EMI SE and power coefficient of absorptivity (A) of 10-2.5 samples with 1 wt.% Fe3O4 nanoparticles after three months of storage at ambient conditions and 10 days of storage in 95% relative humidity environment.

1. **Mechanical Properties**
   1. **Quasistatic Compression Test**


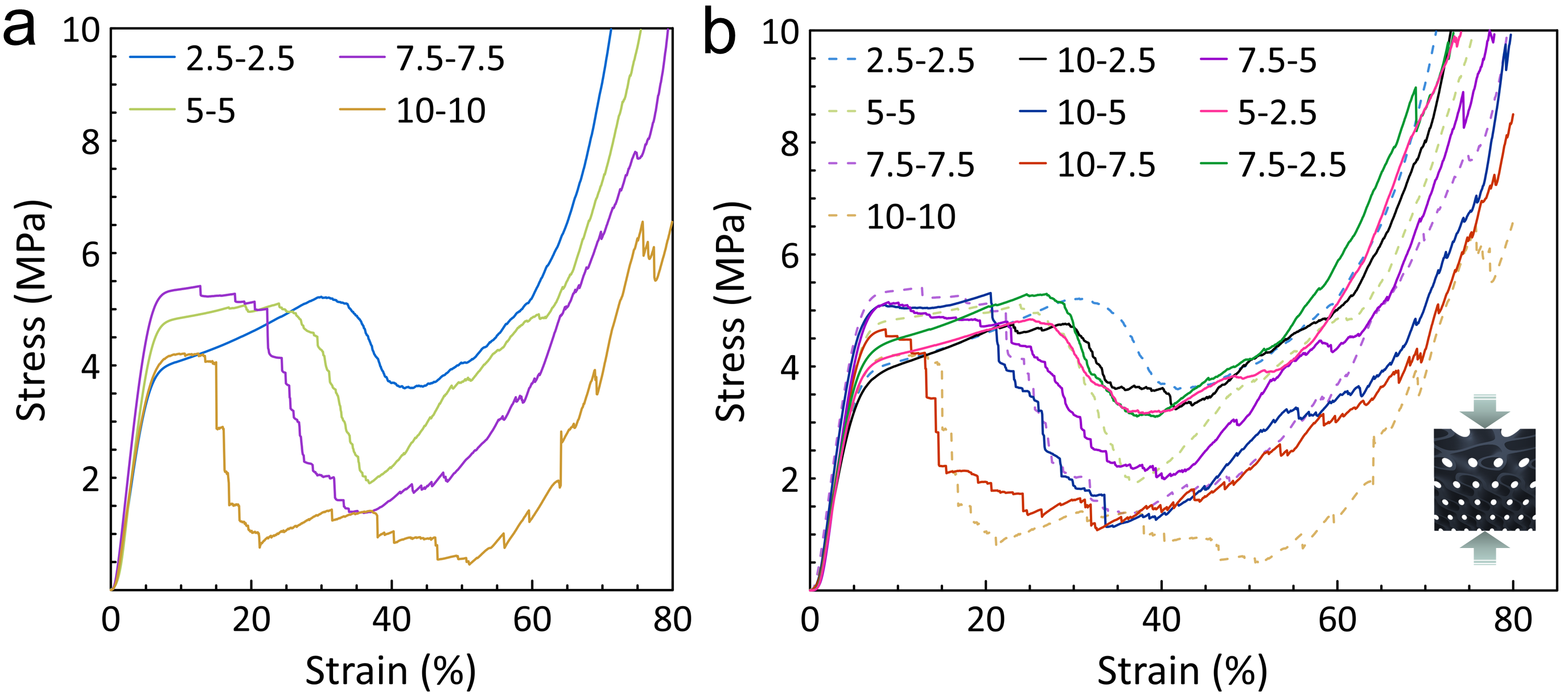


**Fig. S7** Compression stress-strain curves of the gradient conductive Ti_3_C_2_T_x_ MXene-coated TPMS with **a** Uniform and **b** Graded TPMS structure.

- 1. **Cyclic Loading**


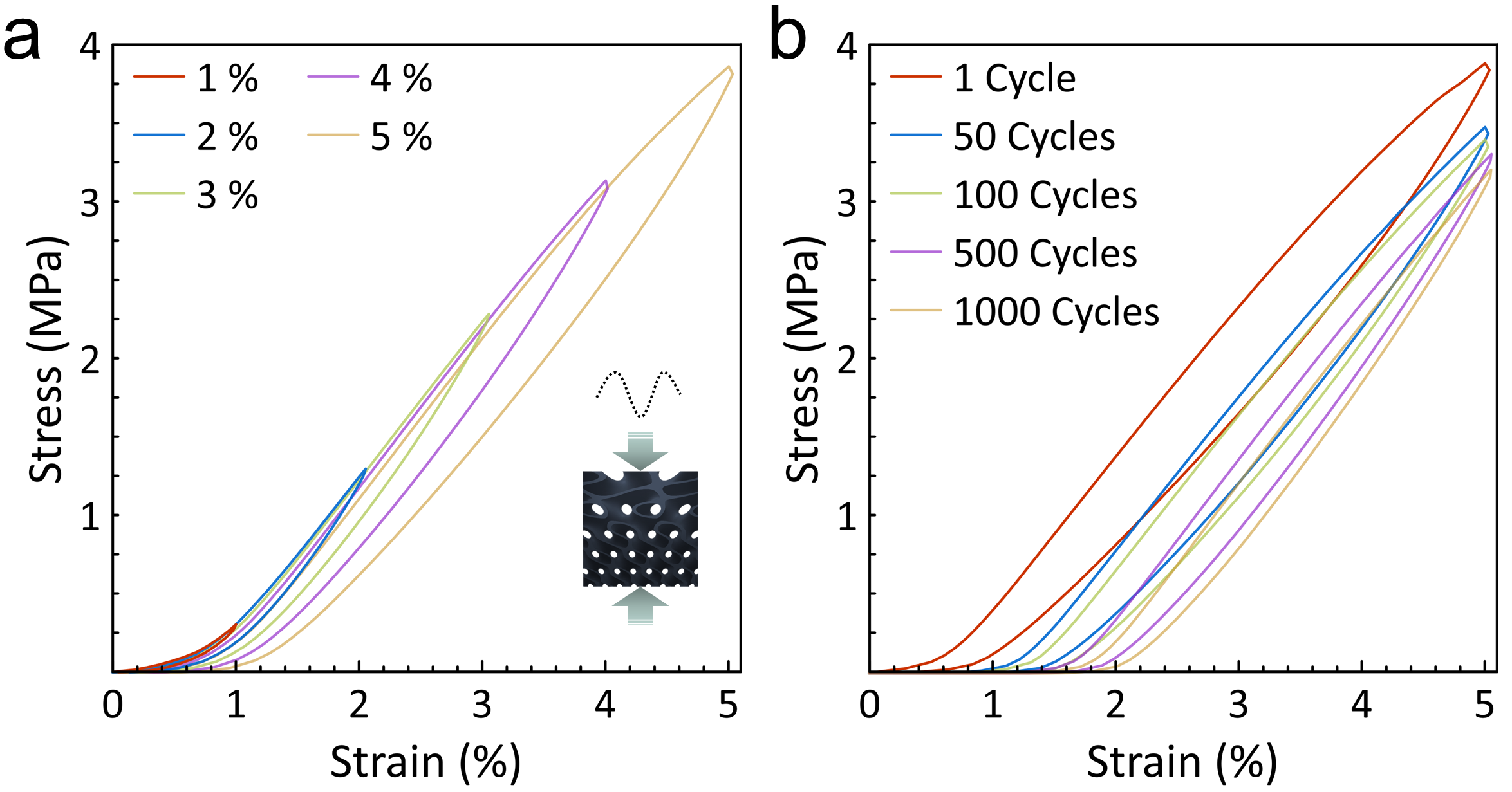


**Fig. S8** Stress-strain curves of the gradient-conductive Ti_3_C_2_T_x_ MXene-coated TPMS structure under cyclic loading.

1. **Comparison with Literature**

**Table S1.** Summary of EMI shielding performance and mechanical properties of the structure developed in this work and structures reported in the literature.

| **Material** | **Thickness (mm)** | **EMI SE_T_ (dB)** | **Absorptivity** | **Strength (MPa)** | **Ref.** |
| --- | --- | --- | --- | --- | --- |
| Graphene-integrated Gyroid TPMS | 15 | ≈ 45-55 | **-** | - | [1] |
| CNT+Fe_3_O_4_-coated Gyroid TPMS | ≈ 10 | 35.9 | 0.87 | ≈ 4 | [2] |
| MXene-functionalized PEDOT:PSS Hydrogel | 0.3 | 51.7 | - | 79 kPa | [3] |
| rGO/PU Foam | 60 | 57.7 | - | - | [4] |
| CNT/PMMA Composite | 4.5 | 40 | - | - | [5] |
| MXene/PS Composite | 2 | 62 | - | - | [6] |
| 3D printed CNT/PLA Composite | 2.5 | 67 | - |  | [7] |
| 3D printed MXene/PLA Composite | 5 | 65 | 0.76 | ≈ 5-6 | [8] |
| MXene-rGO_1:2_ Aerogel | 3 | 50.7 | **-** | 0.4-0.8 kPa | [9] |
| 3D printed MXene/ CNT/PI Aerogel | 5 | 68.2 | 0.77 | ≈ 2-9 | [10] |
| 3D printed GNP/CNT/PLA Composite | 4 | 36.8 | - | - | [11] |
| MWCNT/WPU Foam | 4.5 | 50.5 | - | 20-30 kPa | [12] |
| CF/rGO Aerogel | 5 | 47.8 | - | < 10 kPa | [13] |
| Ti₂CTₓ MXene/PVA Foam | 5 | 28 | 0.59 | - | [14] |
| MXene/ PAM–CMC Hydrogel | 5 | 46.3 | - | 25 kPa | [15] |
| CNF/rGO Aerogel | 5.8 | 65 | 0.68 | - | [16] |
| CNF/APP/Ti_3_C_2_T_x_ Aerogels | 8 | 55 | 0.5 | - | [17] |
| MXene-coated Gyroid TPMS | 20 | 68 | 0.96 | 4-6 | This Work |
| MXene-coated/ 1 wt.% Fe_3_O_4_-integrated Gyroid TPMS | 10 | 59 | 0.85 |  |  |

1. **References**

[1] P. Srinivas, L. Jacob, C. Muhammed Shebeeb, H. Butt, I. Barsoum, R.K. Abu Al-Rub, W. Zaki, Effect of printing parameters and triply periodic minimal surfaces on electromagnetic shielding efficiency of polyvinylidene fluoride graphene nanocomposites, Addit. Manuf. 95 (2024) 104544. https://doi.org/10.1016/j.addma.2024.104544.

[2] C. Wang, Y. Wang, F. Zou, B. Fang, J. Zhao, H. Zhang, J. Guo, L. Jia, D. Yan, Construction of lightweight, high-energy absorption 3D-printed scaffold for electromagnetic interference shielding with low reflection, Compos. Part B Eng. 291 (2025) 112043. https://doi.org/10.1016/j.compositesb.2024.112043.

[3] J. Liu, L. Mckeon, J. Garcia, S. Pinilla, S. Barwich, M. Möbius, P. Stamenov, J.N. Coleman, V. Nicolosi, Additive Manufacturing of Ti 3 C 2 ‐MXene‐Functionalized Conductive Polymer Hydrogels for Electromagnetic‐Interference Shielding, Adv. Mater. 34 (2022) 2106253. https://doi.org/10.1002/adma.202106253.

[4] B. Shen, Y. Li, W. Zhai, W. Zheng, Compressible Graphene-Coated Polymer Foams with Ultralow Density for Adjustable Electromagnetic Interference (EMI) Shielding, ACS Appl. Mater. Interfaces 8 (2016) 8050–8057. https://doi.org/10.1021/acsami.5b11715.

[5] N.C. Das, Y. Liu, K. Yang, W. Peng, S. Maiti, H. Wang, Single‐walled carbon nanotube/poly(methyl methacrylate) composites for electromagnetic interference shielding, Polym. Eng. Sci. 49 (2009) 1627–1634. https://doi.org/10.1002/pen.21384.

[6] R. Sun, H.-B. Zhang, J. Liu, X. Xie, R. Yang, Y. Li, S. Hong, Z.-Z. Yu, Highly Conductive Transition Metal Carbide/Carbonitride(MXene)@polystyrene Nanocomposites Fabricated by Electrostatic Assembly for Highly Efficient Electromagnetic Interference Shielding, Adv. Funct. Mater. 27 (2017) 1702807. https://doi.org/10.1002/adfm.201702807.

[7] Y. Wang, Z.-W. Fan, H. Zhang, J. Guo, D.-X. Yan, S. Wang, K. Dai, Z.-M. Li, 3D-printing of segregated carbon nanotube/polylactic acid composite with enhanced electromagnetic interference shielding and mechanical performance, Mater. Des. 197 (2021) 109222. https://doi.org/10.1016/j.matdes.2020.109222.

[8] S. Lee, D. Kim, N.K. Nguyen, W. Kim, M. Kim, J. Nah, 3D-printed gradient conductivity and porosity structure for enhanced absorption-dominant electromagnetic interference shielding, Carbon N. Y. 231 (2025) 119759. https://doi.org/10.1016/j.carbon.2024.119759.

[9] Z. Fan, D. Wang, Y. Yuan, Y. Wang, Z. Cheng, Y. Liu, Z. Xie, A lightweight and conductive MXene/graphene hybrid foam for superior electromagnetic interference shielding, Chem. Eng. J. 381 (2020) 122696. https://doi.org/10.1016/j.cej.2019.122696.

[10] T. Xue, Y. Yang, D. Yu, Q. Wali, Z. Wang, X. Cao, W. Fan, T. Liu, 3D Printed Integrated Gradient-Conductive MXene/CNT/Polyimide Aerogel Frames for Electromagnetic Interference Shielding with Ultra-Low Reflection, Nano-Micro Lett. 15 (2023) 45. https://doi.org/10.1007/s40820-023-01017-5.

[11] S. Shi, Z. Peng, J. Jing, L. Yang, Y. Chen, 3D Printing of Delicately Controllable Cellular Nanocomposites Based on Polylactic Acid Incorporating Graphene/Carbon Nanotube Hybrids for Efficient Electromagnetic Interference Shielding, ACS Sustain. Chem. Eng. 8 (2020) 7962–7972. https://doi.org/10.1021/acssuschemeng.0c01877.

[12] Z. Zeng, H. Jin, M. Chen, W. Li, L. Zhou, Z. Zhang, Lightweight and Anisotropic Porous MWCNT/WPU Composites for Ultrahigh Performance Electromagnetic Interference Shielding, Adv. Funct. Mater. 26 (2016) 303–310. https://doi.org/https://doi.org/10.1002/adfm.201503579.

[13] Y.-J. Wan, P.-L. Zhu, S.-H. Yu, R. Sun, C.-P. Wong, W.-H. Liao, Ultralight, super-elastic and volume-preserving cellulose fiber/graphene aerogel for high-performance electromagnetic interference shielding, Carbon N. Y. 115 (2017) 629–639. https://doi.org/https://doi.org/10.1016/j.carbon.2017.01.054.

[14] H. Xu, X. Yin, X. Li, M. Li, S. Liang, L. Zhang, L. Cheng, Lightweight Ti2CTx MXene/Poly(vinyl alcohol) Composite Foams for Electromagnetic Wave Shielding with Absorption-Dominated Feature, ACS Appl. Mater. Interfaces 11 (2019) 10198–10207. https://doi.org/10.1021/acsami.8b21671.

[15] Y. Li, X. Xuan, Z. Pan, Y. Li, T.Q. Do, V.H.G. Phan, H. Chen, T. Thambi, A robust and multifunctional conductive double-network hydrogel exhibiting self-healing, anti-freezing, antibacterial, and electromagnetic shielding properties for advanced wearable sensors and biofabrication, Chem. Eng. J. 522 (2025) 167264. https://doi.org/https://doi.org/10.1016/j.cej.2025.167264.

[16] M. Ma, Y. Liao, H. Lin, W. Shao, W. Tao, S. Chen, Y. Shi, H. He, Y. Zhu, X. Wang, Double-layer of CNF/rGO film and CNF/rGO/FeCo-LDO aerogel structured composites for efficient electromagnetic interference shielding, Carbon N. Y. 220 (2024) 118863. https://doi.org/https://doi.org/10.1016/j.carbon.2024.118863.

[17] Y. Zhang, J. Yu, J. Lu, C. Zhu, D. Qi, Facile construction of 2D MXene (Ti3C2Tx) based aerogels with effective fire-resistance and electromagnetic interference shielding performance, J. Alloys Compd. 870 (2021) 159442. https://doi.org/https://doi.org/10.1016/j.jallcom.2021.159442.
